# Hexaamminecobalt(III) Chromate and Dichromate as Precursors of Nanoscale Spinels Co(Co_1−x_Cr_x_)_2_O_4_ (*x* = 0.75; 0.90; 1)

**DOI:** 10.3390/ijms27177914

**Published:** 2026-09-04

**Authors:** Evgeny Filatov, Polina Tarasova, Varvara Sinitsa, Gennady Kostin, Natalia Kuratieva, Pavel Plyusnin, Sergey Korenev

**Affiliations:** Nikolaev Institute of Inorganic Chemistry SB RAS, 3, Acad. Lavrentiev Ave., Novosibirsk 630090, Russiakostin@niic.nsc.ru (G.K.);

**Keywords:** chromite spinel, cobalt, chromium, thermal decomposition

## Abstract

The work develops methods for synthesizing complex salts: [Co(NH_3_)_6_]_2_(CrO_4_)_3_, [Co(NH_3_)_6_](CrO_4_)(NO_3_), [Co(NH_3_)_6_](CrO_4_)Cl·3H_2_O, [Co(NH_3_)_6_]_2_(Cr_2_O_7_)_3_·5H_2_O, [Co(NH_3_)_6_](Cr_2_O_7_)(NO_3_)·H_2_O and [Co(NH_3_)_6_](Cr_2_O_7_)Cl·H_2_O. The compounds were characterized by PXRD, IR and elemental analysis. It was demonstrated that mixed-anion complex salts ([Co(NH_3_)_6_](CrO_4_)(NO_3_) or [Co(NH_3_)_6_](CrO_4_)Cl·3H_2_O) form in the [Co(NH_3_)_6_]^3+^/(CrO_4_)^2−^ system depending on the counterion of hexaamminecobalt(III), [Co(NH_3_)_6_](NO_3_)_3_ or [Co(NH_3_)_6_]Cl_3_, respectively. Conversely, it was more difficult to obtain mixed-anion complexes in the [Co(NH_3_)_6_]^3+^/(Cr_2_O_7_)^2−^ system. Thus, to obtain [Co(NH_3_)_6_](Cr_2_O_7_)Cl·H_2_O an excess of chloride ions in the solution is required. At the same time, when the initial dichromate solution is acidified, the complex [Co(NH_3_)_6_]_2_(Cr_2_O_7_)_3_·5H_2_O is formed, exhibiting structural polymorphism: the crystal structure changes without altering the chemical composition when stored in a closed container. The process of thermal decomposition of all synthesized complex compounds in an inert atmosphere has been studied. The final product of the thermolysis of most compounds at a temperature of 600 °C is a nanoscale single-phase solid solution Co(Co_1−x_Cr_x_)_2_O_4_ with a spinel structure (with crystallite sizes of no more than 15 nm), where the metal ratio corresponds to that of the initial complex salt. The smallest crystallite size (3–4 nm) was obtained through the thermolysis of [Co(NH_3_)_6_](CrO_4_)Cl.

## 1. Introduction

Coordination compounds are widely used as molecular precursors for the preparation of multicomponent inorganic materials because they enable different metal species to be combined at the molecular or atomic scale before thermal treatment [1,2]. In addition, some coordination compounds themselves exhibit useful functional properties [3,4]. Of particular interest in precursor chemistry are double complex salts (DCS), i.e., ionic compounds containing both cationic and anionic coordination entities with identical or different central metal atoms [5,6,7]. The close spatial proximity of different metals in such compounds can facilitate the formation of finely dispersed bimetallic products upon thermolysis.

Thermal decomposition of coordination precursors provides access to a broad range of nanoscale materials, including solid solutions, intermetallic compounds and mixed oxides. The composition and phase state of the resulting products can be controlled by the precursor composition as well as by thermolysis conditions such as atmosphere, heating rate or final decomposition temperature. Consequently, coordination compounds have attracted considerable attention as precursors for materials exhibiting catalytic [8], magnetic [9] and other functional properties [10,11]. An additional advantage of ammine complexes is that decomposition of the ammonia-containing coordination sphere occurs at relatively moderate temperatures, which may favor the formation of highly dispersed products.

The hexaamminecobalt(III) chromate and dichromate salts considered in the present work are not DCS in the strict coordination-chemical sense because only the cobalt atom is incorporated into a conventional coordination sphere, [Co(NH_3_)_6_]^3+^. Nevertheless, these compounds contain Co and Cr species homogeneously combined within a single-crystalline precursor and are therefore of interest for precursor chemistry. Their thermal decomposition can potentially provide a direct route to nanosized cobalt–chromium oxide phases while preserving, to a significant extent, the metal ratio established in the initial compound. This makes the [Co(NH_3_)_6_]^3+^/(CrO_4_)^2−^ and [Co(NH_3_)_6_]^3+^/(Cr_2_O_7_)^2−^ systems particularly suitable for studying the relationship between precursor composition, thermal behavior and the composition of the resulting oxide phases.

Cobalt–chromium oxide systems are of interest because of their diverse structural and physical properties. In particular, nanoscale CoCr_2_O_4_ spinel exhibits unusual magnetic behavior [12,13], while magnetic and structural effects in Co-Cr_2_O_3_ composite systems have also been actively investigated [14,15]. The use of molecular precursors can be advantageous for preparing such materials because Co and Cr are already mixed on the molecular scale. A related example is the double complex salt [Co(NH_3_)_6_][Cr(C_2_O_4_)_3_], whose thermal decomposition under oxidizing and reducing conditions has previously been studied [16]. Depending on the atmosphere, its thermolysis was shown to produce Co and Cr_2_O_3_ mixtures or mixed cobalt–chromium oxides, including CoCr_2_O_4_ and Co_2_CrO_4_ [16]. These results demonstrate that coordination compounds containing both Co and Cr can serve as effective precursors for compositionally controlled oxide materials.

Despite this potential, the chemistry of hexaamminecobalt(III) salts with chromate and dichromate anions remains insufficiently explored. To date, only two compounds in the [Co(NH_3_)_6_]^3+^—(CrO_4_)^2−^/(Cr_2_O_7_)^2−^ systems have been structurally characterized: [Co(NH_3_)_6_](CrO_4_)Cl·3H_2_O [17] and [Co(NH_3_)_6_](Cr_2_O_7_)Cl·H_2_O [18]. Consequently, the influence of the chromate/dichromate anion, additional outer-sphere anions and the Co:Cr ratio on the crystal structures and thermal decomposition products of these compounds has not been systematically established.

In the present work, we address this gap by synthesizing and characterizing a series of hexaamminecobalt(III) chromate and dichromate compounds: [Co(NH_3_)_6_](CrO_4_)Cl·3H_2_O (**I**), [Co(NH_3_)_6_](CrO_4_)(NO_3_) (**II**), [Co(NH_3_)_6_]_2_(CrO_4_)_3_ (**III**), [Co(NH_3_)_6_](Cr_2_O_7_)Cl·H_2_O (**IV**), [Co(NH_3_)_6_](Cr_2_O_7_)(NO_3_)·H_2_O (**V**), [Co(NH_3_)_6_]_2_(Cr_2_O_7_)_3_·5H_2_O (**VI**). Their compositions and crystal structures were investigated using elemental analysis, IR spectroscopy, powder X-ray diffraction (PXRD) and single-crystal X-ray diffraction (SCXRD). Particular attention was paid to their thermal decomposition in an inert atmosphere and to the identification and characterization of the resulting Co–Cr oxide phases. The aim was to establish how the composition of the molecular precursor affects thermal stability, phase formation and the composition and crystallite size of the resulting nanoscale spinel-type Co(Co_1−x_Cr_x_)_2_O_4_ products.

## 2. Results and Discussion

### 2.1. Synthesis Features

As is well known, chromate and dichromate anions are in chemical equilibrium in an aqueous solution: 2CrO_4_^2−^ + 2H^+^ ⇌ Cr_2_O_7_^2−^ + H_2_O. Accordingly, during the synthesis of hexaamminecobalt(III) dichromates, the solutions were acidified, and during the synthesis of chromates, the pH was increased by adding concentrated ammonia.

In the case of hexaamminecobalt(III) chromate synthesis from the [Co(NH_3_)_6_]Cl_3_ more stable form, [Co(NH_3_)_6_](CrO_4_)Cl·3H_2_O was obtained. However, when attempting synthesis from the precursor [Co(NH_3_)_6_](NO_3_)_3_, which does not contain chloride ions, crystals of a new previously unknown compound were formed—[Co(NH_3_)_6_](CrO_4_)(NO_3_). To obtain the target compound ([Co(NH_3_)_6_]_2_(CrO_4_)_3_), silver oxide was added to the hexaamminecobalt(III) chloride solution and the resulting AgCl precipitate was filtered off. This left only hexaamminecobalt(III) hydroxide in the solution to which a chromate solution was then added. Only in this case was it possible to obtain a precipitate, which, according to PXRD data, is isostructural to the anhydrous [Cr(NH_3_)_6_]_2_(CrO_4_)_3_ [19]. TG and elemental data also confirmed that the compound is anhydrous. At the same time, it was possible to grow single crystals from the mother liquor, which according to SCXRD data, were [Co(NH_3_)_6_]_2_(CrO_4_)_3_·5H_2_O with a structure that differs from that of the anhydrous salt.

**Figure 1:**
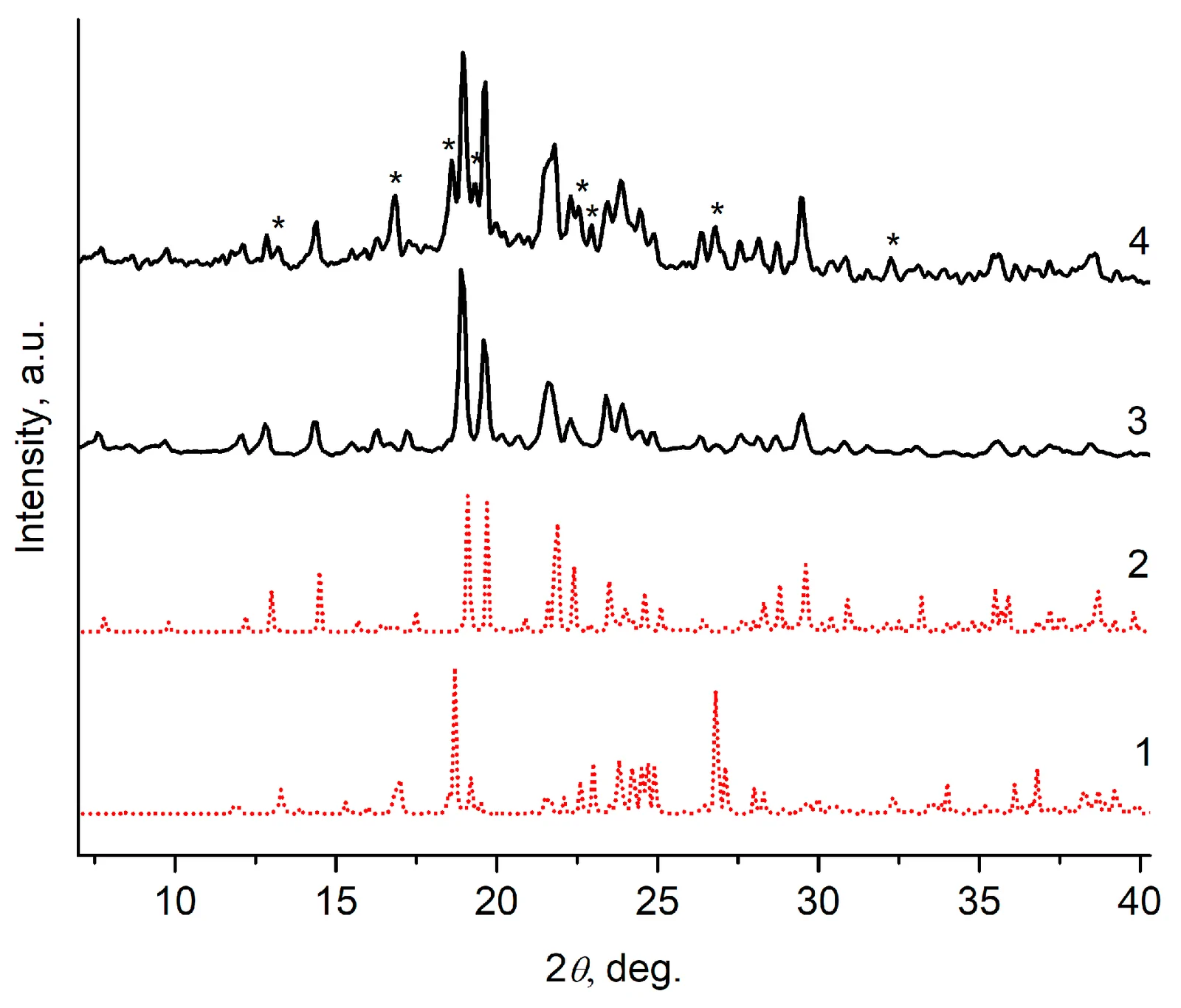
1 and 2—XRD patterns obtained from the single-crystal data for [Co(NH_3_)_6_]_2_(Cr_2_O_7_)_3_·5H_2_O with space groups *P*2_1_/*c* and *P*2_1_/*n*, respectively. 3 and 4—powder XRD patterns for freshly precipitated [Co(NH_3_)_6_]_2_(Cr_2_O_7_)_3_·5H_2_O and for the same sample after 4 months of storage in a closed container. * marks the peaks corresponding to the phase with the *P*2_1_/*c* space group.

In the case of dichromate-containing salt synthesis, however, simple mixing of K_2_Cr_2_O_7_ and [Co(NH_3_)_6_]Cl_3_ solutions did not result in a single-phase product of hexaamminecobalt(III) dichromate chloride as it was stated in the method [18]. The precipitate always contained a significant amount of [Co(NH_3_)_6_]_2_(Cr_2_O_7_)_3_·*n*H_2_O. It was only possible to obtain [Co(NH_3_)_6_](Cr_2_O_7_)Cl·H_2_O by increasing the concentration of chloride ions in the solution by adding KCl (up to 2/3 equivalent of cobalt). When the initial dichromate solution was acidified, the complex [Co(NH_3_)_6_]_2_(Cr_2_O_7_)_3_·5H_2_O formed, which crystallizes in a monoclinic crystal system (space group *P*2_1_/*n*). It should be noted that this structure is metastable: when the compound is stored in a closed container for a long time the crystal structure changes. After four months, peaks (~30%) corresponding to another structure of the pentahydrate hexaamminecobalt(III) dichromate (space group *P*2_1_/*c*) appear along with peaks of [Co(NH_3_)_6_]_2_(Cr_2_O_7_)_3_·5H_2_O (space group *P*2_1_/*n*) (Figure 1).

During the synthesis of hexaamminecobalt(III) dichromate using chloride-free [Co(NH_3_)_6_](NO_3_)_3_, crystals of a previously unknown structure, [Co(NH_3_)_6_](Cr_2_O_7_)(NO_3_)·H_2_O were formed.

### 2.2. Complex Salts Crystal Structures and IR Spectra

The key structural units of the studied complex compounds are octahedral [Co(NH_3_)_6_]^3+^ cations and chromate or dichromate anions. The main geometric parameters of these fragments are given in Table 1. In all the described compounds, the [Co(NH_3_)_6_]^3+^ cation is a slightly distorted octahedron with central angles differing from 90° by no more than 1.7°. The Co-N bond lengths are in the range of 1.95–1.98 Å which is typical for Co^3+^ complexes with ammonia ligands. The change from chromate anions (**II** and **III**) to dichromate anions (**V** and **VI**) results in a slight decrease in Cr-O_term_ bond lengths (0.02–0.04 Å). Moreover, the Cr-O bridge bonds in dichromate anions are consistently longer. The Cr-O-Cr angle at the bridging oxygen atom varies within a fairly wide range (117.9–130.4°) depending on the packing. All structures contain an extended network of hydrogen bonds involving ammonia ligands of cation complexes, and terminal oxygen atoms of chromate or dichromate anions, as well as solvate water molecules or nitrate ions with a D-A distance of 2.80 to 3.06 Å.

All the obtained compounds contain the [Co(NH_3_)_6_]^3+^ cation; therefore, all the IR spectra exhibit bands associated with N–H vibrations of coordinated ammonia (Figure S1, Table S1). The stretching vibrations *ν*_as_(N–H) occur in the 3150–3265 cm^−1^ region, while the *ν*_s_(N–H) vibrations are observed in the 3125–3165 cm^−1^ region. The N–H deformation vibrations appear in the 1600–1650 and 1330–1360 cm^−1^ regions. The vibrations of coordinated ammonia occur at lower frequencies than those of free NH_3_, which can be attributed to the coordination effect and interactions with the counterions (CrO_4_^2−^, Cr_2_O_7_^2−^, Cl^−^ and NO_3_^−^). This shift may be associated with the weakening of the N–H bonds due to the formation of N–H···Cl and N–H···O hydrogen bonds [20]. Chromate and dichromate ions can be clearly distinguished in the low-frequency region. Thus, bands in the 825–890 cm^−1^ region are characteristic of CrO_4_^2−^, whereas a more complex set of bands is observed in the 730–940 cm^−1^ region for Cr_2_O_7_^2−^. In hydrated salts, crystallization water is indicated by broad bands in the 3385–3565 cm^−1^ region. The 700–950 cm^−1^ region is particularly informative for identifying the anion, as the characteristic band patterns differ significantly between chromates and dichromates. In addition, replacement of Cl^−^ by NO_3_^−^ is reflected in the 1330–1360 cm^−1^ region, where the nitrate ion contributes to the observed absorption. Thus, the N–H region is similar for all the salts due to the common [Co(NH_3_)_6_]^3+^ cation, whereas the differences in their IR spectra are mainly determined by the nature of the anion (CrO_4_^2−^ or Cr_2_O_7_^2−^), the presence of NO_3−_ and crystallization water.

#### 2.2.1. [Co(NH_3_)_6_]_2_(CrO_4_)_3_·5H_2_O (**III**) Crystal Structure

**Figure 2:**
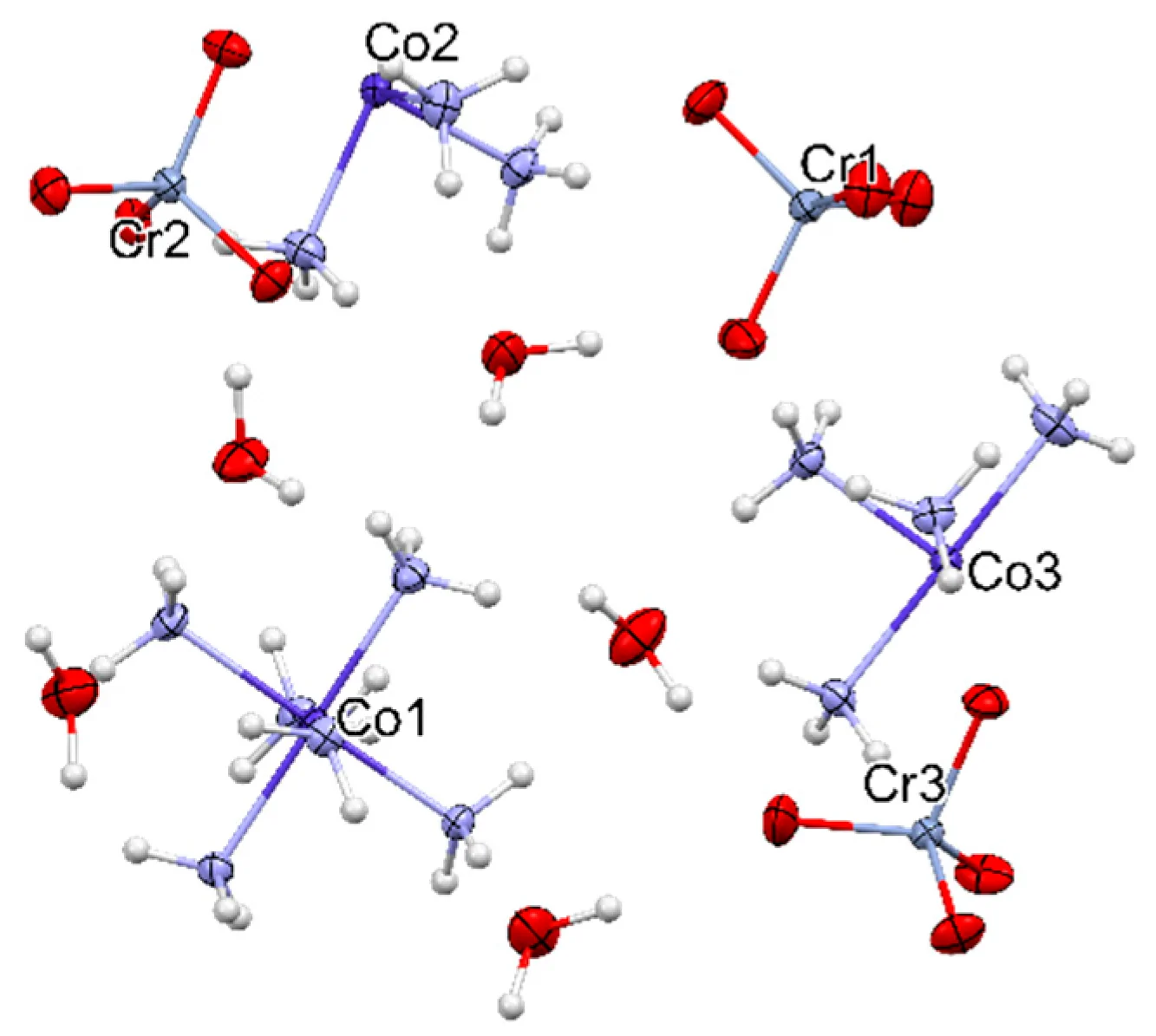
Fragment of the [Co(NH_3_)_6_]_2_(CrO_4_)_3_·5H_2_O structure in ellipsoid representation (50% probability).

**Figure 3:**
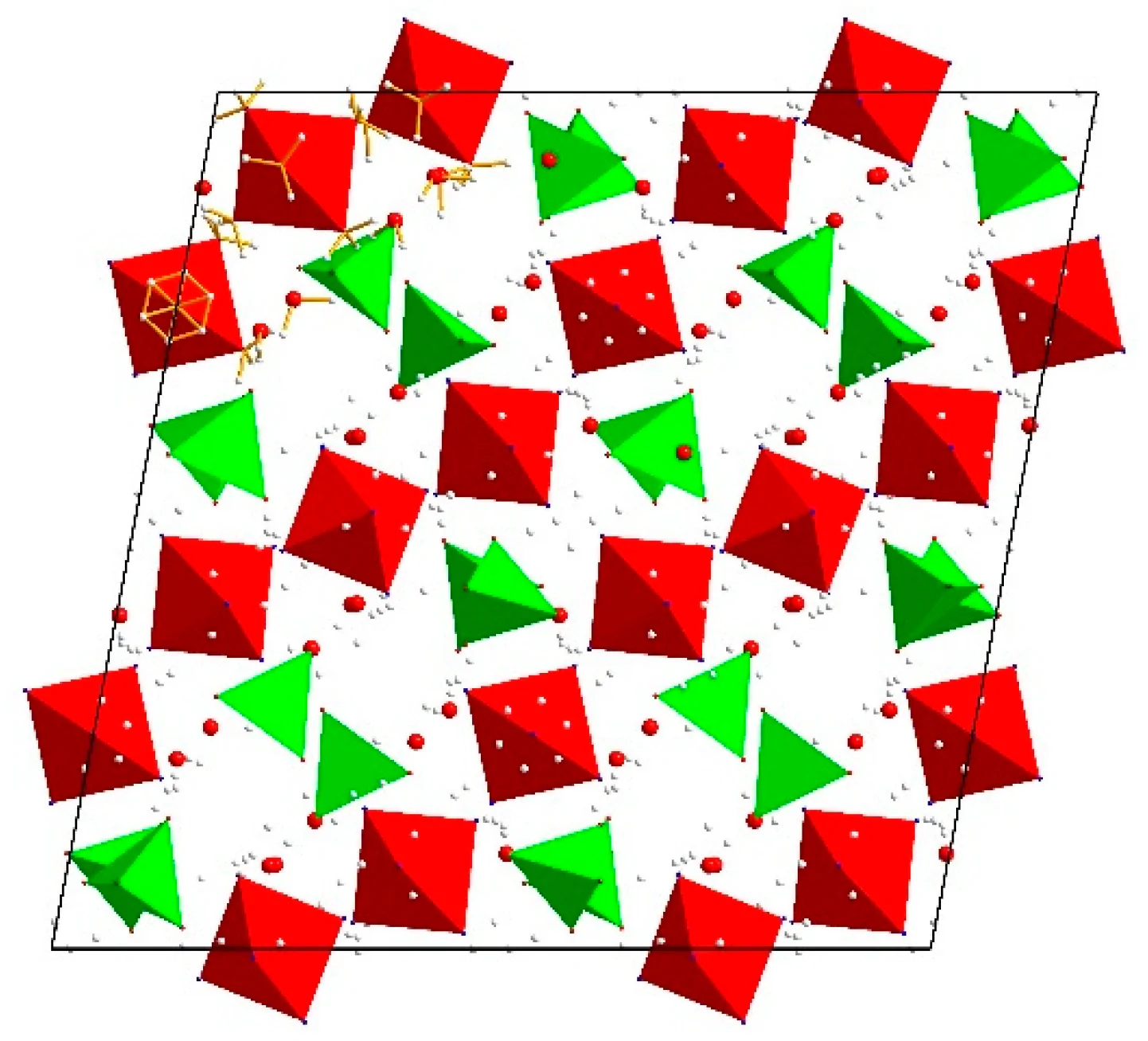
Cation and anion layers in the [Co(NH_3_)_6_]_2_(CrO_4_)_3_·5H_2_O structure, packing view along *b*. The green color shows the {CrO_4_} tetrahedra and the red color shows the {CoN_6_} octahedra.

The complex salt crystallizes in the *C*2/*c* space group. All chromate anions are located in common positions while complex cations occupy one common position with single occupancy and two special positions (1/4 1/4 0 and 0 *y* 1/4) with position occupations of 0.50/0.50. The crystal structure contains five water molecules per formula unit [Co(NH_3_)_6_]_2_(CrO_4_)_3_ (Figure 2). The packing can be represented as an alternation of loose cation and anion layers that are parallel to the *ac* plane (Figure 3). The cation and anion sublattices are connected by a system of hydrogen bonds involving crystallization water molecules. In all figures the following color designation of atoms is used: gray—chromium, violet—cobalt, red—oxygen, blue—nitrogen, white—hydrogen.

#### 2.2.2. [Co(NH_3_)_6_](CrO_4_)(NO_3_) (**II**) Crystal Structure

**Figure 4:**
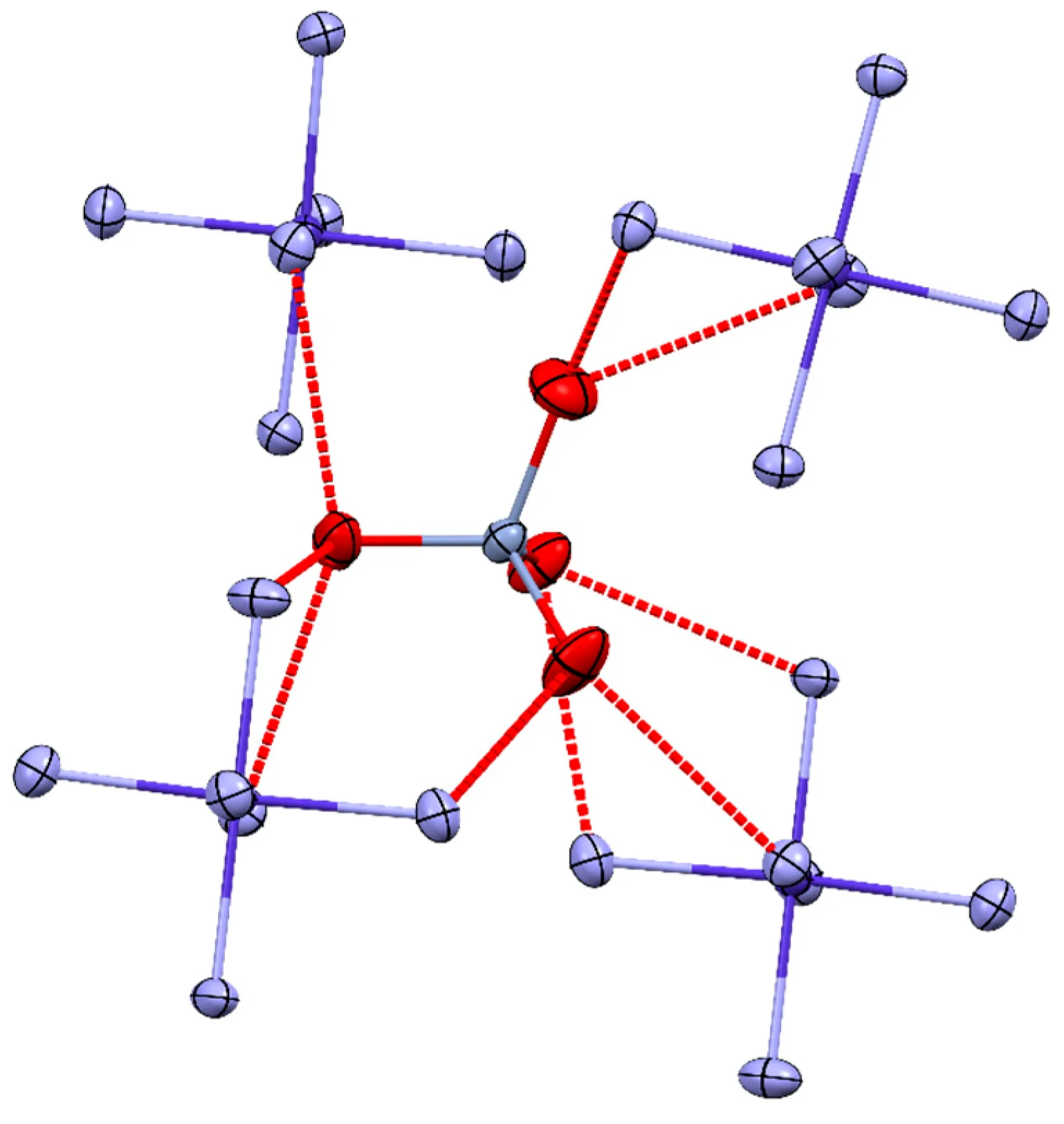
Fragment of the [Co(NH_3_)_6_](CrO_4_)(NO_3_) structure in ellipsoid representation (50% probability). The red lines are used to indicate hydrogen bonds.

The compound crystallizes in the *I*4_1_*/acd* space group. Chromate anions are located in common positions while the complex cations occupy two special positions (0 0 *z*) and (*x x* 1/4) with a 1/2 occupancy. The chromate anions are in a tetrahedral environment of four neighboring complex cations. Hydrogen bonds form between the oxygen atoms of CrO_4_^2−^ and hydrogen atoms of the NH_3_ ligands with N…O distances in the range of 2.863–2.975 Å (Figure 4).

#### 2.2.3. [Co(NH_3_)_6_]_2_(Cr_2_O_7_)_3_·5H_2_O (**VI**) Crystal Structure

As mentioned above, two polymorphic modifications of hexaamminecobalt(III) dichromate pentahydrate (**VI_1_**) and (**VI_2_**) have been identified.

**Figure 5:**
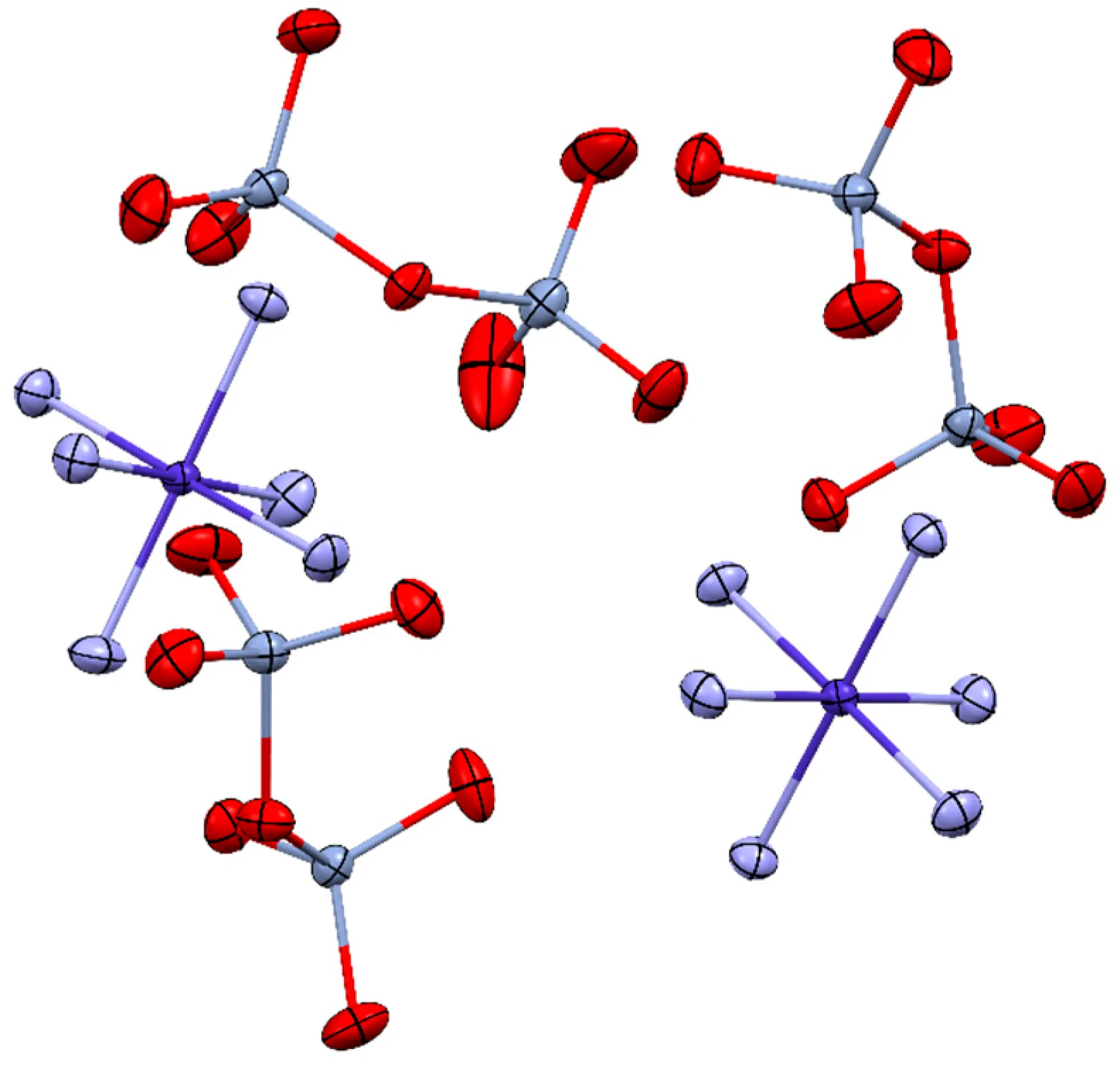
Fragment of the [Co(NH_3_)_6_]_2_(Cr_2_O_7_)_3_·5H_2_O (**VI_1_**) structure in ellipsoid representation (50% probability).

The metastable polymorphic modification **VI_1_** crystallizes in the *P*2_1_/*n* space group with a unit cell volume of 3360.2 Å^3^ (*Z* = 4). The asymmetric unit of the structure includes two structurally independent cations, three dichromate anions, and five water molecules, two of which are disordered between two positions (Figure 5). One dichromate anion has a conformation close to eclipsed with O-Cr-Cr-O torsion angles in the range of 8–10° while the other two dichromate anions have torsion angles of 25–35°.

**Figure 6:**
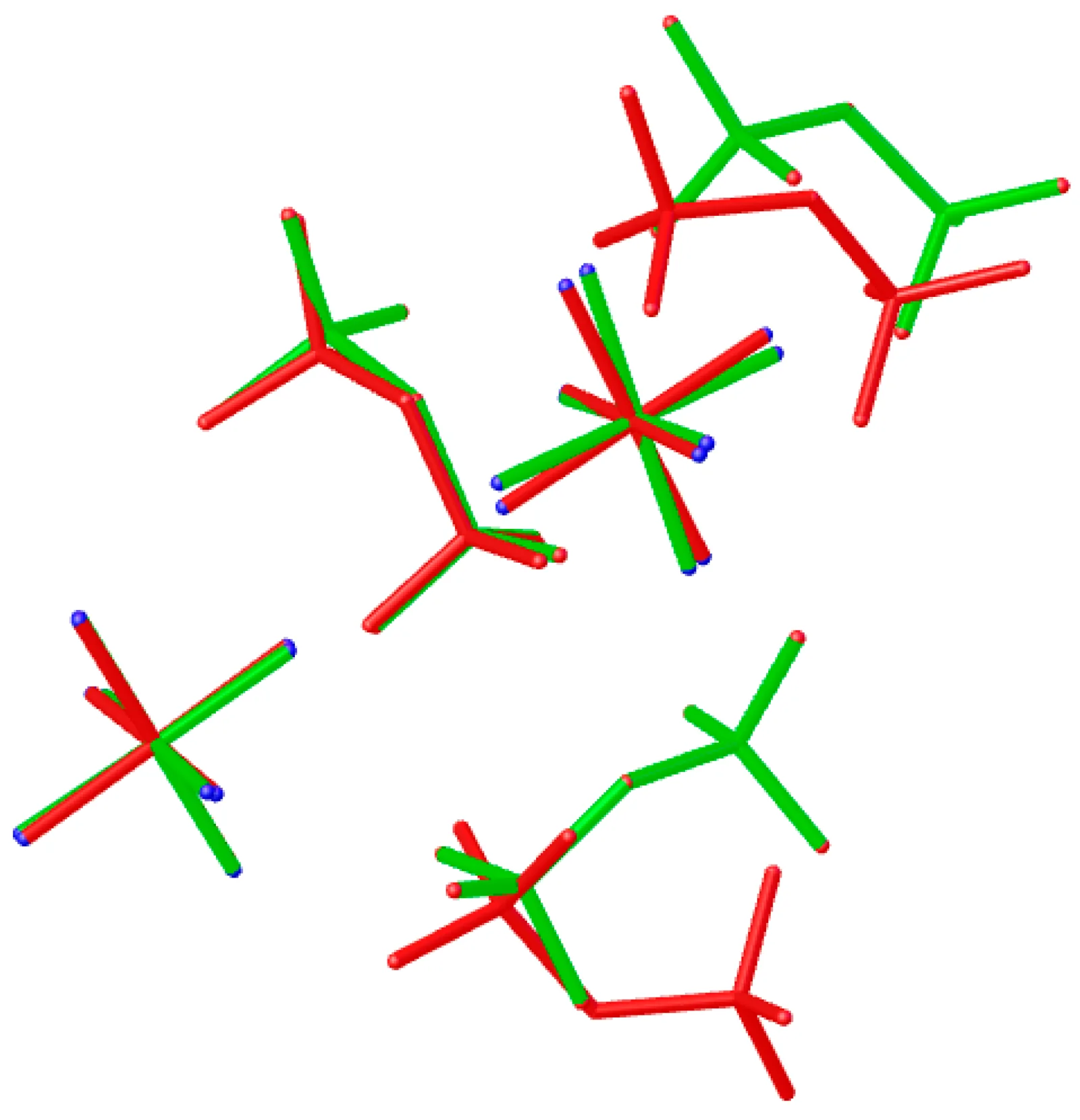
[Co(NH_3_)_6_]_2_(Cr_2_O_7_)_3_·5H_2_O polymorphic modifications **VI_1_** (red) and **VI_2_** (green).

Stable **VI_2_** modification, which has the same composition as **VI_1_**, crystallizes in the *P*2_1_/*c* space group with a cell volume of 3391.1 Å^3^ (*Z* = 4). Complex cations are slightly distorted octahedra. Two dichromate anions have a conformation close to eclipsed with torsion angles ranging from 11.3 to 13.8°. One anion has a conformation close to staggered with torsion angles of 63.2–67.4°. Comparing the asymmetric fragments of the two polymorphic modifications reveals that the most significant change in position occurs with the dichromate anions, one of which rotates during the modification change (Figure 6).

#### 2.2.4. [Co(NH_3_)_6_](Cr_2_O_7_)(NO_3_)·H_2_O (**V**) Crystal Structure

**Figure 7:**
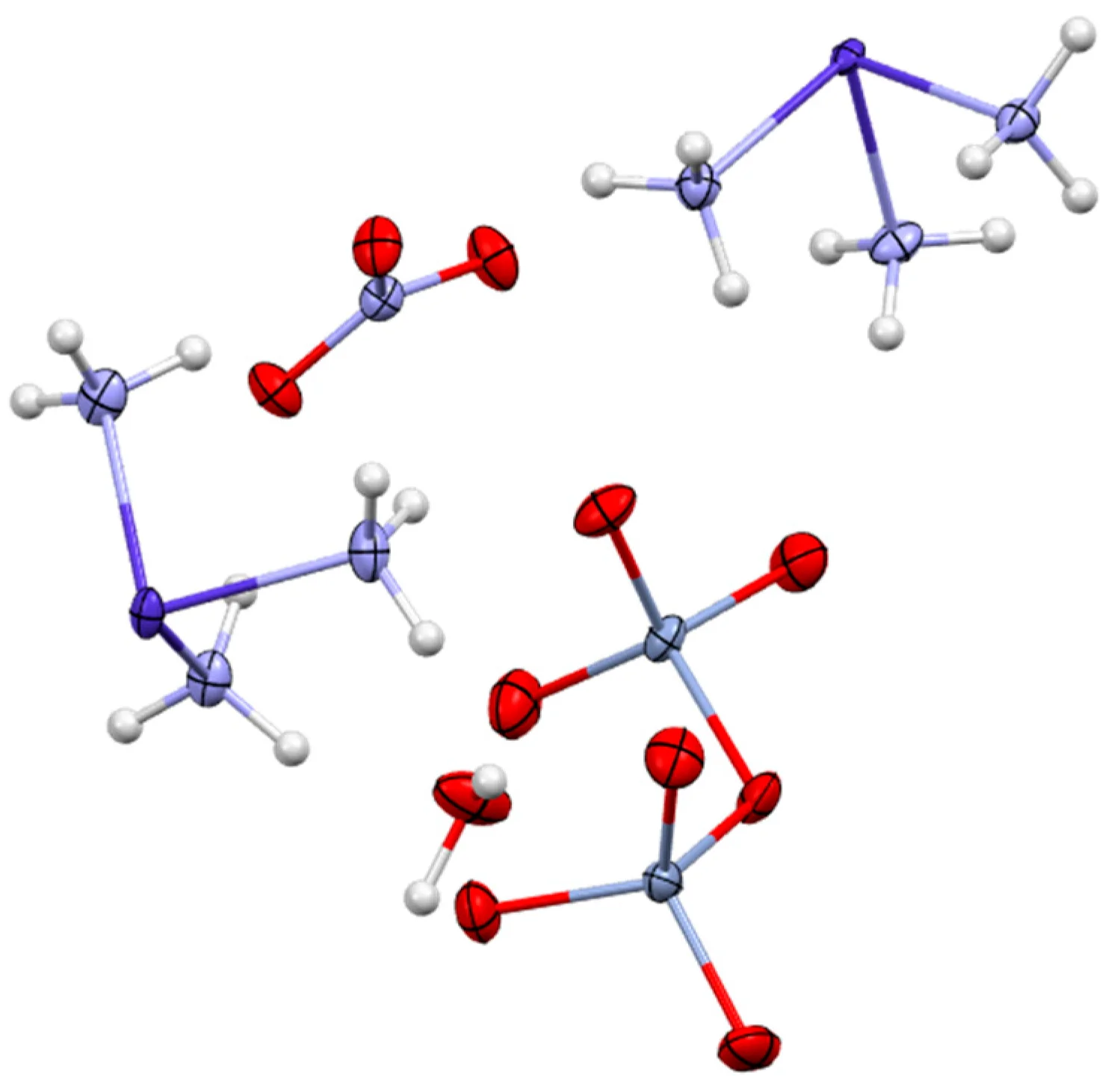
Fragment of the [Co(NH_3_)_6_](Cr_2_O_7_)(NO_3_)·H_2_O (**V**) structure in ellipsoid representation (50% probability).

The asymmetric fragment of the structure involves two structurally independent cations, [Co(NH_3_)_6_]^3+^, located in the special positions (0 0 1/2) and (0 1/2 0) with occupancies of 1/2, a dichromate anion, a nitrate anion and one water molecule (Figure 7). The dichromate anion is in an eclipsed conformation with O_term_-Cr-Cr-O_term_ torsion angles ranging from 8 to 9°. The mutual arrangement of the complex cobalt cations and dichromate anions within the structure can be described as a distorted NaCl-type structure with six counterions located near each ion. All nitrogen atoms of the complex cations as well as oxygen atoms of the anions (dichromate and nitrate) and water molecules participate in hydrogen bonds with a D-A distance ranging from 2.80 to 3.06 Å.

**Figure 8:**
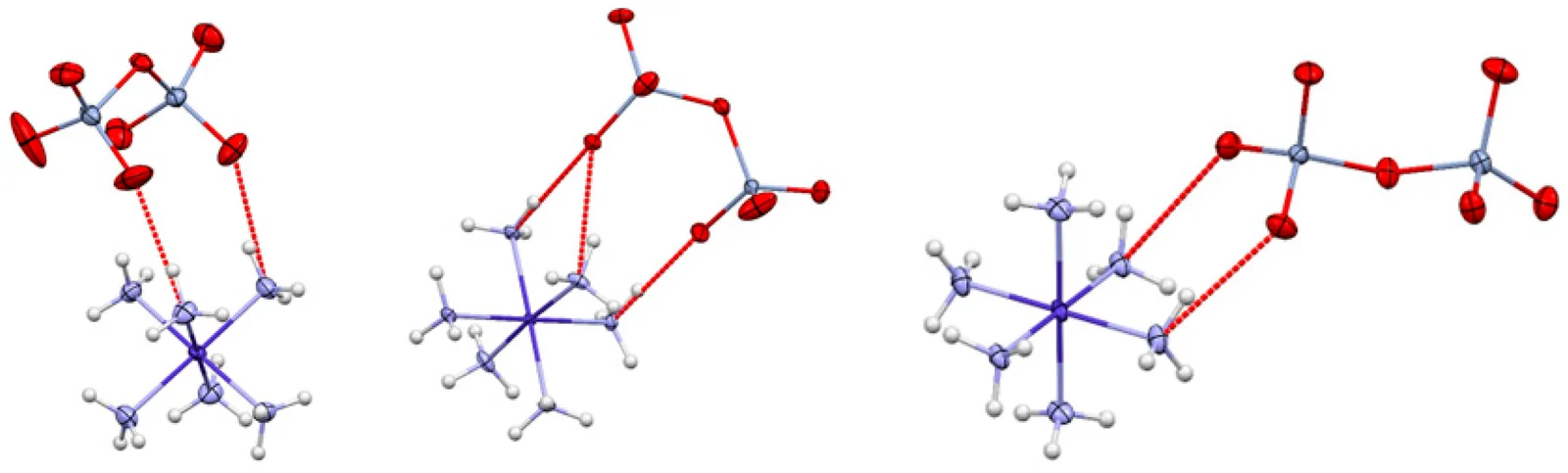
Various types of hydrogen bonds between dichromate anions and [Co(NH_3_)_6_]^3+^ cations in structures **V** and **VI**.

Comparing the hydrogen bonds in structures with dichromate anions (**V**–**VI**), two main types of anion-cation hydrogen bonds can be distinguished in these structures. In one case, two terminal atoms from different “CrO_4_” tetrahedra bond to two (or three) ammonia ligands of the cation; in the second case, two hydrogen bonds with one cation are formed by two oxygen atoms from one “CrO_4_” fragment (Figure 8). The bridging oxygen atoms of dichromate anions do not participate in hydrogen-bond formation.

The crystal frameworks in the investigated compounds are formed by the hydrogen bonds of different types. Total lists of H-bonds are given in Supplementary Materials (Tables S2–S6). In all compounds the main framework is formed by the cation-anion interactions. Hirshfeld surfaces for cations are given in Supplementary Materials (Table S7) and formed only by the interactions of NH_3_ hydrogen atoms, with specific H-bond interactions forming 65–87% of Hirshfeld surfaces. Other hydrogen bonds important for the investigated compound frameworks are listed in Table 2. Solvate-water clusters with H_2_O-H_2_O hydrogen bonds are present in structures **VI_1_** and **VI_2_**. In structure **V,** despite the large number of solvate water molecules, these molecules are separated and bound mainly to chromate anions.

### 2.3. Thermolysis of Complex Salts

**Figure 9:**
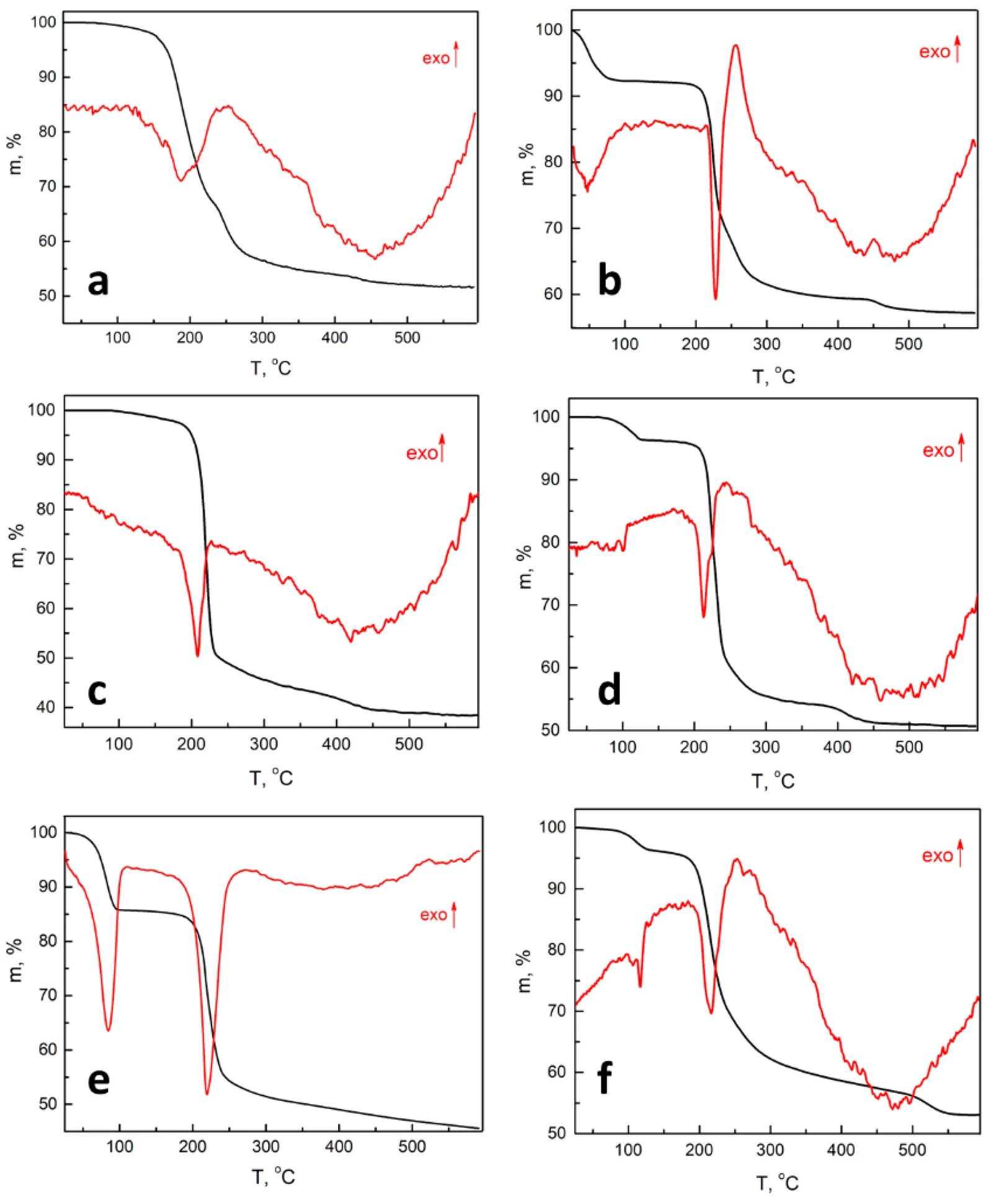
Thermal analysis curves (TG—black line, DTA—red line) of synthesized compounds in an inert atmosphere: (**a**) [Co(NH_3_)_6_]_2_(CrO_4_)_3_ (**III**), (**b**) [Co(NH_3_)_6_]_2_(Cr_2_O_7_)_3_·5H_2_O (**VI**), (**c**) [Co(NH_3_)_6_](CrO_4_)(NO_3_) (**II**), (**d**) [Co(NH_3_)_6_](Cr_2_O_7_)(NO_3_)·H_2_O (**V**), (**e**) [Co(NH_3_)_6_](CrO_4_)Cl·3H_2_O (**I**), (**f**) [Co(NH_3_)_6_](Cr_2_O_7_)Cl·H_2_O (**IV**).

Thermogravimetric analysis was used to study the thermal stability and decomposition characteristics of the synthesized compounds in an inert (helium) atmosphere at temperatures ranging from 25 to 600 °C (Figure 9).

When the compound contained crystallization water, a mass loss corresponding to the dehydration process was observed. Further decomposition begins with the destruction of the cobalt coordination sphere followed by the thermal destruction of the anionic part. The mass loss stages in the TG curves for almost all anhydrous complexes are poorly separated. This means that various processes overlap; for example, the end of the ammonia evolution stage coincides with the beginning of the oxygen evolution stage. Where possible, the corresponding values are given.

The thermal decomposition of [Co(NH_3_)_6_]_2_(CrO_4_)_3_ (**III**) proceeds in three poorly separated steps (Figure 9a) accompanied by a transition from an endo effect to an exo effect and a second endo effect, which is extended over the temperature range. In the first step (54–220 °C) a loss of 30.5% (204 Da) is observed corresponding to the removal of all the ammonia ligands. In the second step (220–400 °C) a loss of 16% (107 Da) occurs. The product obtained by thermolysis up to 350 °C followed by cooling is X-ray amorphous. The third step of decomposition (400–600 °C) is accompanied by a 2.5% (17 Da) loss in mass. According to PXRD data, the final product of thermolysis of hexaamminecobalt(III) chromate at 600 °C is a single-phase product with a spinel structure. Based on the metal ratio in the initial compound (Co:Cr = 2:3) this product can be attributed to the composition Co_1.2_Cr_1.8_O_4_ or Co^II^(Co_0.1_Cr_0.9_)_2_^III^O_4_. Refinement of the unit cell parameter by the full-profile method yields a value of *a* = 8.302(5) Å, which is in good agreement with the linear dependence of the unit cell parameter on the spinel composition. The average crystallite size is 10–12 nm.

The thermal decomposition of [Co(NH_3_)_6_](CrO_4_)(NO_3_) (**II**) proceeds similarly to the decomposition of “pure” hexaamminecobalt(III) chromate except that only two poorly separated steps can be distinguished on the TG curve (Figure 9c). The first step (90–330 °C) is accompanied by an endothermic effect and a 56% mass loss. In the second step (330–570 °C) the mass loss is 5.5%. It is not possible to calculate unequivocally from these data which products go into the gas phase. However, according to PXRD data, the product obtained by heating to 350 °C followed by cooling contains a spinel phase, which corresponds to the Co_2_CrO_4_ phase (*a* = 8.17(1) Å, PDF #24-326). The product obtained at 600 °C also contains a spinel phase which, according to the unit cell parameter (*a* = 8.270(5) Å), already corresponds to one with a higher chromium content. Based on the metal ratio in the initial compound (Co:Cr = 1:1), the final product of thermolysis can be attributed to the composition Co_1.5_Cr_1.5_O_4_ or Co^II^(Co_0.25_Cr_0.75_)_2_^III^O_4_. The average crystallite size is 9–12 nm.

Thermolysis of [Co(NH_3_)_6_](CrO_4_)Cl·3H_2_O (**I**) occurs in several steps (Figure 9e) accompanied by endo effects. At the first step (27–110 °C), a loss of 14.7% (54 Da) is observed, corresponding to the removal of three water molecules. At the second step (133–600 °C), a loss of 43.3% (159 Da) occurs, corresponding to the removal of all the ammonia molecules and part of the oxygen. The final products of thermolysis at 600 °C are a spinel phase with a unit cell parameter of *a* = 8.324(5) Å (which corresponds to the composition CoCr_2_O_4_) and a Co_2_(OH)_3_Cl phase. The average crystallite size is 3–4 nm. Relatively stable cobalt(II) chloride is apparently formed during thermolysis and does not decompose until 600 °C.

A comparison of the thermal decomposition curves of hexaamminecobalt(III) chromate (**III**) (Figure 9a) and dichromate (**VI**) (Figure 9b) compounds shows how the nature of the anion affects the thermal decomposition process. The less stable salt with the chromate anion begins to decompose slowly at temperatures of ~55 °C, whereas the anhydrous complex with the dichromate anion remains relatively stable, only beginning to lose mass at around 120 °C. During the decomposition of hexaamminecobalt(III) dichromate (**VI**), a significant loss of mass (30%) is observed in the temperature range from 175 °C to 280 °C, accompanied by a transition from an endothermic effect to an exothermic one. The intermediate decomposition products are X-ray amorphous. Thermolysis is completed at a temperature of 590 °C. According to PXRD data and quantitative Rietveld refinement, the final product is a mixture of CoCr_2_O_4_ (78(3) wt.%) and Cr_2_O_3_ (22(3) wt.%) (average crystallite size 10–13 nm), which corresponds within the error limits to the initial metal ratio Co:Cr = 2:3.

In the case of the decomposition of [Co(NH_3_)_6_](Cr_2_O_7_)(NO_3_)·H_2_O (**V**) and [Co(NH_3_)_6_](Cr_2_O_7_)Cl·H_2_O (**IV**), the first step (30–130 °C) proceeds with an endo effect (Figure 9d,f) and is accompanied by the removal of crystallization water. Further decomposition of the anhydrous salts as for [Co(NH_3_)_6_](Cr_2_O_7_)(NO_3_) (**V**) proceeds in two poorly separated steps with the onset of thermolysis shifting towards higher temperatures (140–585 °C). According to PXRD data, the product of thermolysis of both complex salts at 600 °C is a CoCr_2_O_4_ spinel with an average crystallite size of 5–8 nm.

Table 3 summarizes data on the thermolysis of complex salts. As can be seen, complex salts with dichromate anions are more thermally stable than those with chromate anions. The presence of nitrate or chloride anions in the outer sphere increases the stability of anhydrous complex compounds. If the ratio of metals in the initial complex compound does not exceed Co:Cr = 1:2, a single-phase solid solution based on cochromite spinel Co^II^(Co_1−x_Cr_x_)_2_^III^O_4_ with crystallite sizes of no more than 15 nm is formed. However, if the chromium content in the initial compound exceeds the cobalt content by more than two times, the excess chromium is separated as a Cr_2_O_3_ phase. It should be noted that the spinel phase with the smallest crystallite size (5–8 nm) forms during the thermolysis of [Co(NH_3_)_6_](Cr_2_O_7_)(NO_3_) (**V**) possibly due to the relative stability of the intermediate products of thermolysis.

**Figure 10:**
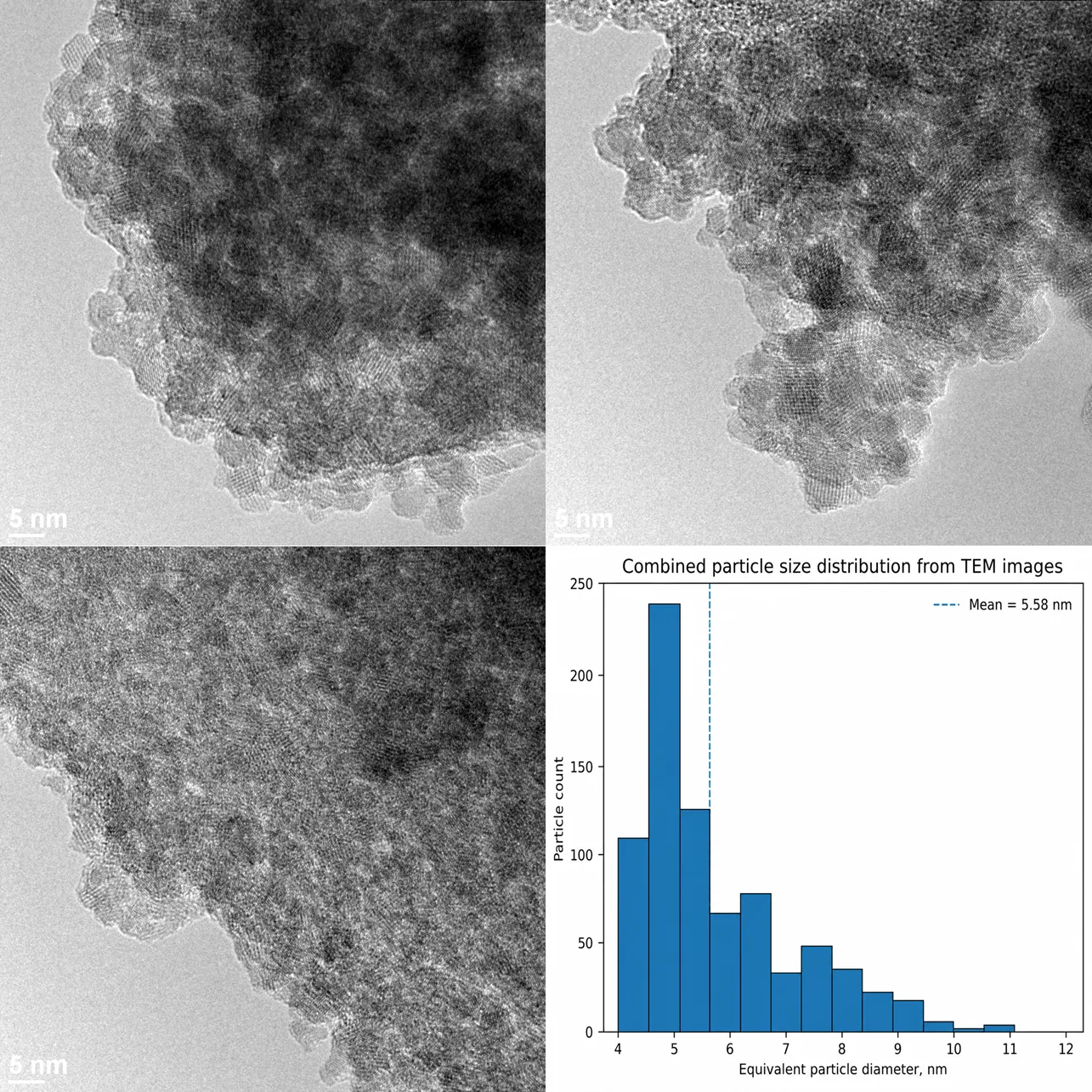
HRTEM images of the thermolysis product [Co(NH_3_)_6_](Cr_2_O_7_)(NO_3_), obtained in an inert atmosphere at 600 °C, and a histogram of the particle size distribution.

To confirm the agreement between the crystallite size, estimated from the PXRD data using the Scherrer equation, and the particle size of the thermolysis products, a high-resolution transmission electron microscopy (HRTEM) study was carried out on a single-phase product with a spinel structure, which has the smallest calculated crystallite sizes. Figure 10 shows an example of spinel obtained by thermolysis of [Co(NH_3_)_6_](Cr_2_O_7_)(NO_3_).

Analysis of a series of TEM images revealed the formation of predominantly finely dispersed nanoparticles with similar size characteristics in different regions of the sample. Based on the processing of a set of 766 particles, the average equivalent diameter was 5.6 ± 1.3 nm, and the median value was 5.1 nm. The sizes of the recorded particles were predominantly in the range of several nanometers, with an overall range of 3.3–8.9 nm. The average values determined from individual TEM images varied within a narrow range of 5.3–5.8 nm, indicating a relatively uniform size distribution of nanoparticles in the studied regions of the sample. The presence of a small proportion of larger particles leads to a moderate right-hand asymmetry in the distribution.

## 3. Materials and Methods

### 3.1. Materials

Initial compounds [Co(NH_3_)_6_]Cl_3_ and [Co(NH_3_)_6_](NO_3_)_3_ were synthesized by known methods [21]. Commercially available (NH_4_)_2_CrO_4_, (NH_4_)_2_Cr_2_O_7_, K_2_Cr_2_O_7_, Ag_2_O, KCl, KOH were used. The purity of all reagents was 99.5%.

### 3.2. Synthesis of [Co(NH_3_)_6_](CrO_4_)Cl·3H_2_O *(**I**)*

A total of 0.3993 g of [Co(NH_3_)_6_]Cl_3_ was dissolved in 5 mL of distilled water. Separately, 0.3747 g of ammonium chromate (NH_4_)_2_CrO_4_ was dissolved in 4 mL of concentrated aqueous ammonia solution. The hexaamminecobalt(III) complex solution was then added to the ammonium chromate solution with stirring, resulting in the immediate formation of a yellow crystalline precipitate. The reaction mixture was then cooled in a freezer to increase the completeness of the precipitation. The precipitate was then filtered and washed with ethanol. Yield is 93%. Elemental analysis, found/calc. for CoN_6_H_24_CrO_7_Cl (%): N 23.0/22.92; H 6.2/6.60. IR spectroscopy (KBr, cm^−1^): 3400 − *ν*(O–H), 3250_wide_ − *ν*_as_(N–H), 3152 − *ν*_s_(N–H), 1650 − *δ*_d_(N–H), 1330 − *δ*_s_(N–H), 860 − *ν*(Cr–O) + *ρ*_r_(N–H).

### 3.3. Synthesis of [Co(NH_3_)_6_](CrO_4_)(NO_3_) *(**II**)*

A total of 0.5180 g of [Co(NH_3_)_6_](NO_3_)_3_ was dissolved in 25 mL of distilled water. Due to the limited solubility of the salt, the process was carried out with stirring and heating to 60 °C under a watch glass. At the same time, 0.3750 g of ammonium chromate (NH_4_)_2_CrO_4_ was dissolved in 5 mL of concentrated ammonia. The cooled cobalt complex solution was then added to the chromate solution. A yellow precipitate formed instantly. The precipitate was filtered and washed with ethanol. Yield is 97%. Elemental analysis, found/calc. for CoN_7_H_18_CrO_7_ (%): N 28.2/28.91; H 5.4/5.35. IR spectroscopy (KBr, cm^−1^): 3160_wide_ − *ν*_as_(N–H) + *ν*_s_(N–H), 1600 − *δ*_d_(N–H), 1360 − *δ*_s_(N–H) + *ν*(N–O), 870 − *ν*(Cr–O) + *ρ*_r_(N–H).

### 3.4. Synthesis of [Co(NH_3_)_6_]_2_(CrO_4_)_3_ *(**III**)*

A batch of Ag_2_O (0.9151 g) was added to a solution of 0.6408 g of [Co(NH_3_)_6_]Cl_3_ in 10 mL of distilled water. The mixture was stirred for 3 h after which the AgCl precipitate was filtered off. A solution of 0.5806 g of K_2_Cr_2_O_7_ alkalized with KOH to a pH of ~10 was then added to the filtrate. The fine crystalline precipitate was then filtered and washed with ethanol. Yield is 97%. Elemental analysis, found/calc. for Co_2_N_12_H_36_Cr_3_O_12_ (%): N 24.6/25.08; H 5.7/5.41. IR spectroscopy (KBr, cm^−1^): 3150_wide_ − *ν*_as_(N–H) + *ν*_s_(N–H), 1600 − *δ*_d_(N–H), 1350 − *δ*_s_(N–H), 890 − *ν*(Cr–O), 825 − *ρ*_r_(N–H).

### 3.5. Synthesis of [Co(NH_3_)_6_](Cr_2_O_7_)Cl·H_2_O *(**IV**)*

A hot solution of K_2_Cr_2_O_7_ (0.1095 g in 1 mL of aqueous hydrochloric acid solution (pH ≈ 4)) was added to a solution containing 0.1000 g of [Co(NH_3_)_6_]Cl_3_ and 0.0188 g of KCl in 3 mL of distilled water at a temperature close to boiling. Almost immediately, an orange precipitate formed. The reaction mixture was slowly cooled to the room temperature and then stirred on a magnetic stirrer for 24 h. The precipitate was filtered and washed with ethanol. Yield is 31%. Elemental analysis, found/calc. for CoN_6_H_20_Cr_2_O_8_Cl (%): N 19.6/19.52; H 4.5/4.68. IR spectroscopy (KBr, cm^−1^): 3510+3385 − *ν*(O–H), 3255 − *ν*_as_(N–H), 3125 − *ν*_s_(N–H), 1610 − *δ*_d_(N–H), 1330 − *δ*_s_(N–H), 940 − *ν*(Cr–O), 875 − *ρ*_r_(N–H), 750 − *ν*(Cr–O–Cr).

### 3.6. Synthesis of [Co(NH_3_)_6_](Cr_2_O_7_)(NO_3_)·H_2_O *(**V**)*

A total of 0.3580 g of [Co(NH_3_)_6_](NO_3_)_3_ was dissolved in 20 mL of distilled water with stirring on a magnetic stirrer at 60 °C. In a separate beaker, a solution of ammonium dichromate was prepared: 0.4288 g of (NH_4_)_2_Cr_2_O_7_ was dissolved in 5 mL of diluted perchloric acid (HClO_4_, pH ≈ 4). The cobalt complex solution was then added to the resulting dichromate solution. A yellow flocculent precipitate formed immediately. To increase the completeness of the precipitation, the reaction mixture was kept in a freezer. The precipitate was filtered and washed with ethanol. Yield is 99%. Elemental analysis, found/calc. for CoN_7_H_20_Cr_2_O_11_ (%): N 21.1/21.45; H 4.4/4.41. IR spectroscopy (KBr, cm^−1^): 3565 + 3430 − *ν*(O–H), 3265 − *ν*_as_(N–H), 3165 − *ν*_s_(N–H), 1620 − *δ*_d_(N–H), 1350 − *δ*_s_(N–H) + *ν*(N–O), 930 − *ν*(Cr–O), 880 − *ρ*_r_(N–H), 835 − *ν*(N–O), 730 − *ν*(Cr–O–Cr).

### 3.7. Synthesis of [Co(NH_3_)_6_]_2_(Cr_2_O_7_)_3_·5H_2_O *(**VI**)*

A total of 0.798 g of [Co(NH_3_)_6_]Cl_3_ was dissolved in 90 mL of distilled water. In a separate beaker, 1.3200 g of K_2_Cr_2_O_7_ was dissolved in a mixture of 10 mL of distilled water and 5 mL of 1.73 M HCl. The cobalt complex solution was then added to the dichromate solution. A yellow flocculent precipitate formed, which was filtered and washed with ethanol. Yield is 82%. Elemental analysis, found/calc. for Co_2_N_12_H_46_Cr_6_O_26_ (%): N 16.7/15.85; H 3.9/4.37. IR spectroscopy (KBr, cm^−1^): 3485 − *ν*(O–H), 3250 − *ν*_as_(N–H), 3160 − *ν*_s_(N–H), 1605 − *δ*_d_(N–H), 1335 − *δ*_s_(N–H), 940 − *ν*(Cr–O), 875 − *ρ*_r_(N–H), 730 − *ν*(Cr–O–Cr). SCXRD data show that the complex compound may have two polymorphic modifications with the space groups *P*2_1_/*n* (**VI_1_**) and *P*2_1_/*c* (**VI_2_**).

Single crystals of all the compounds were obtained by slow evaporation of mother liquors in air. Powder XRD data of complex compounds confirmed the single-phase nature of the products. The identity of single-crystal samples with the bulk product for all compounds except for [Co(NH_3_)_6_]_2_(CrO_4_)_3_ was confirmed by the complete agreement of the theoretical diffraction pattern calculated from single-crystal XRD data with the powder diffraction pattern. In the case of [Co(NH_3_)_6_]_2_(CrO_4_)_3_ the alignment of single-crystal samples to the main mass of the product was confirmed by the complete coincidence between the theoretical XRD pattern calculated from single-crystal XRD data for the previously described [Cr(NH_3_)_6_]_2_(CrO_4_)_3_ [19] and the experimentally recorded powder XRD pattern. PXRD and IR-spectroscopy data of the synthesized complex compounds are given in the Supplementary Materials (Figures S1–S3).

### 3.8. Characterization

**Powder XRD**. Powder X-ray diffraction (PXRD) studies of complex salts were done in the 2*θ* range 5–60° and thermolysis products of the prepared compound were determined in the 2θ range 5–90° on a HAOYUAN DX-2700BH diffractometer (CuK_α_-radiation, graphite monochromator on the diffracted beam and ambient temperature). Thermolysis products were identified by analogy with X-ray diffraction patterns of pure metals and oxides according to the PDF database [22]. The powder diffraction patterns were analyzed by Rietveld refinement using the PowderCell 2.4 program [23]. The diffraction patterns of the thermolysis products of the synthesized compounds and their simulation of the corresponding phases using the Rietveld method are shown in Table S8. Determination of the spinel Co(Co_1−x_Cr_x_)_2_O_4_ compositions was based on additive dependence of the cell parameter (*a*) on Cr content using calibration curves of *a* versus *x* (*a*—cell parameter Å, *x *— Cr content in octahedral position). Calibration curves were based on the literature data [24,25,26,27,28,29,30,31,32,33,34,35,36] (Figure S4). The crystallite sizes of the oxide phases were determined using the Scherrer equation (WINFIT 1.2.1) [37].

**Single-crystal X-ray diffraction** (SCXRD) data for synthesized compounds were collected using the graphite monochromatized MoK_α_-radiation (*λ* = 0.71073 Å) at 150(2) K on a X8APEX Bruker Nonius diffractometer (Bruker AXS, Karlsruhe, Germany) equipped with a 4K CCD area detector. The *φ*-scan technique was employed to measure intensities. Absorption correction was applied empirically using the SADABS program [38]. The structure was solved by the direct methods of the difference Fourier synthesis and further refined by the full-matrix least squares method using the SHELXTL package v.2018/3 [39]. Atomic thermal parameters for non-hydrogen atoms were refined anisotropically. The positions of hydrogen atoms of ammonia ligands were calculated corresponding to their geometrical conditions and refined using the riding model, while the hydrogen atom positions of co-crystallized water molecules were localized from the difference maps and refined in the isotropic approximation with constraining of the O-H distance to about 0.96(1) Å. The structural data have been deposited into CCDC with the numbers 2533411–2533415. Crystallographic data and details of the single-crystal XRD experiment for the studied compounds are given in Tables S9 and S10 (Supplementary Materials).

**Elemental analysis** was made with the Vario Micro Cube (Elementar, Langenselbold, Germany) automatic analyzer of the analytical laboratory of NIIC SB RAS.

**IR spectroscopy.** IR spectra (400–4000 cm^−1^) of powders in KBr pellets were recorded using a Scimitar FTS 2000 FTIR spectrometer (Digilab LLC, Randolph, MA, USA) [40].

**Thermal analysis.** Thermogravimetric analyses (TGA) were carried out on a TG 209 F1 Iris thermobalance (NETZSCH, Waldkraiburg, Germany). The simultaneous thermogravimetry and differential scanning calorimetry (TG-DTA) measurements were performed using a STA 449 F1 Jupiter thermal analyzer. The measurements were made using Al crucibles with sample masses ranging from 10 to 50 mg in helium flow in the temperature range of 30–600 °C (without annealing) at a heating rate of 10 K/min and a gas flow rate of 60 mL/min. The treatment of experimental results was performed using standard software, Proteus analysis.

**High-resolution transmission electron microscopy** (HRTEM) images were obtained with a JEM-2200FS transmission electron microscope (JEOL Ltd., Tokyo, Japan, acceleration voltage 200 kV, lattice resolution—1 Å) equipped with a Cs-corrector. The samples for the TEM study were prepared by ultrasonic dispersion in ethanol followed by deposition of the suspension upon a “holey” carbon film supported on a copper grid.

## 4. Conclusions

This study presents methods for synthesizing [Co(NH_3_)_6_]_2_(CrO_4_)_3_, [Co(NH_3_)_6_](CrO_4_)(NO_3_), [Co(NH_3_)_6_](CrO_4_)Cl·3H_2_O, [Co(NH_3_)_6_]_2_(Cr_2_O_7_)_3_·5H_2_O, [Co(NH_3_)_6_](Cr_2_O_7_)(NO_3_)·H_2_O and [Co(NH_3_)_6_](Cr_2_O_7_)Cl·H_2_O. The compounds were characterized by PXRD, SCXRD, IR spectroscopy and elemental analysis. Mixed-anion complex salts have been shown to form in the [Co(NH_3_)_6_]^3+^/(CrO_4_)^2−^ system, with the type of salt formed depending on the counterion of hexaamminecobalt(III): nitrate, chromate or chloride chromate. In order to obtain [Co(NH_3_)_6_]_2_(CrO_4_)_3_ it is necessary to first remove chloride anions from hexaamminecobalt(III) chloride. In contrast, obtaining mixed-anion complexes with dichromate ions proved more difficult. For the synthesis of [Co(NH_3_)_6_](Cr_2_O_7_)Cl·H_2_O, an excess of chloride ions in the solution is required. Acidifying the initial dichromate solution led to the [Co(NH_3_)_6_]_2_(Cr_2_O_7_)_3_·5H_2_O formation, which crystallizes in a monoclinic crystal system (space group *P*2_1_/*n*). This compound exhibits structural polymorphism: when stored in a closed container for a long time the crystal structure changes without altering the chemical composition. The thermal decomposition process of all synthesized complex compounds in an inert atmosphere has been studied. When a compound contains crystallization water, a loss of mass is observed, corresponding to the removal of water molecules. Complex salts containing dichromate anions are more thermally stable than those containing chromate anions. The presence of nitrate or chloride anions in the outer sphere increases the stability of anhydrous complex compounds. The final product of thermolysis of most compounds at 600 °C is a nanoscale single-phase solid solution of Co(Co_1−x_Cr_x_)_2_O_4_ with a spinel structure (with crystallite sizes not exceeding 15 nm) where the metal ratio corresponds to that in the precursor compound. Exceptions are the thermolysis product of [Co(NH_3_)_6_]_2_(Cr_2_O_7_)_3_, where excess chromium is released as a second phase—Cr_2_O_3_; and the thermolysis product of [Co(NH_3_)_6_](CrO_4_)Cl, where a Co_2_(OH)_3_Cl phase forms alongside a spinel phase. The smallest crystallite size (3–4 nm) was obtained by thermolysis of [Co(NH_3_)_6_](CrO_4_)Cl. The single-phase product CoCr_2_O_4_ (5–8 nm) with the smallest crystallite size was obtained by thermolysis of the [Co(NH_3_)_6_](Cr_2_O_7_)(NO_3_). The ability to obtain nanosized spinels of various compositions, Co(Co_1−x_Cr_x_)_2_O_4_, can be used in the future for obtaining stable and highly effective catalysts.

## Figures and Tables

**Table 1:** Geometric parameters of [Co(NH_3_)_6_](CrO_4_)(NO_3_) (**II**), [Co(NH_3_)_6_]_2_(CrO_4_)_3_·5H_2_O (**III)**, [Co(NH_3_)_6_](Cr_2_O_7_)(NO_3_)·H_2_O (**V**) and [Co(NH_3_)_6_]_2_(Cr_2_O_7_)_3_·5H_2_O with space group *P*2_1_/*n* (**VI_1_**) and *P*2_1_/*c* (**VI_2_**).

	II	III	V	VI_1_	VI_2_
d(Co-N), Å	1.969(2)–1.974(2)	1.957(2)–1.977(2)	1.964(2)–1.974(2)	1.955(2)–1.971(2)	1.957(4)–1.981(3)
∠ N-Co-N, °	88.1(1)–91.3(1)	87.8(1)–91.7(1)	89.2(1)–90.8(1)	88.1(1)–91.6(1)	89.1(1)–90.7(2)
d(Cr-O_term_), Å	1.641(2)–1.651(2)	1.632(2)–1.669(2)	1.613(1)–1.621(1)	1.588(3)–1.629(2)	1.585(4)–1.616(3)
d(Cr-O_br_), Å	–	–	1.774(1)–1.801(1)	1.780(2)–1.799(2)	1.772(3)–1.794(3)
∠ Cr-O-Cr, °	–	–	119.9(1)	117.9(1)–124.5(1)	121.5(2)–130.4(2)

**Table 2:** Donor–acceptor distance (Å) in hydrogen bonds in investigated structures [Co(NH_3_)_6_](CrO_4_)(NO_3_) (**II**), [Co(NH_3_)_6_]_2_(CrO_4_)_3_·5H_2_O (**III**), [Co(NH_3_)_6_](Cr_2_O_7_)(NO_3_)·H_2_O (**V**) and [Co(NH_3_)_6_]_2_(Cr_2_O_7_)_3_·5H_2_O with space group *P*2_1_/*n* (**VI_1_**) and *P*2_1_/*c* (**VI_2_**).

	II	III	V	VI_1_	VI_2_
NH_3_-O(Cr)	2.923–3.095	2.913–3.092	2.963–3.098	2.886–3.083	2.894–3.094
NH_3_-O(H_2_O)	-	2.861–3.095	3.063	2.887–3.069	2.944–3.076
NH_3_-O(NO_3_)	2.948–3.073	-	2.927–3.011	-	-
(Cr)O-O(H_2_O)	-	2.663–3.056	2.809–3.035	2.946–3.096	2.832–3.032
O(H_2_O)-O(H_2_O)	-	-	-	2.827–2.886	2.790–2.814

**Table 3:** Summarized data on the thermal decomposition of the complex salts: the temperature range of the decomposition of anhydrous compounds as well as the data after refinement by the Rietveld method of the thermolysis products obtained in an inert atmosphere at a temperature of 600 °C.

Precursor	Thermolysis Temperature Range, °C	Unit Cell Parameter, Å	Composition	Crystallite Size, nm
(**I**) [Co(NH_3_)_6_](CrO_4_)Cl	133–600	8.324(5)	CoCr_2_O_4_ + Co_2_(OH)_3_Cl	3–4
(**II**) [Co(NH_3_)_6_](CrO_4_)(NO_3_)	90–600	8.270(5)	Co_1.5_Cr_1.5_O_4_	9–12
(**III**) [Co(NH_3_)_6_]_2_(CrO_4_)_3_	54–600	8.302(5)	Co_1.2_Cr_1.8_O_4_	10–12
(**IV**) [Co(NH_3_)_6_](Cr_2_O_7_)Cl	140–585	8.326(4)	CoCr_2_O_4_	8–13
(**V**) [Co(NH_3_)_6_](Cr_2_O_7_)(NO_3_)	165–585	8.324(4)	CoCr_2_O_4_	5–8
(**VI**) [Co(NH_3_)_6_]_2_(Cr_2_O_7_)_3_	120–590	8.324(4)	CoCr_2_O_4_ + Cr_2_O_3_	10–13

## Data Availability

The original contributions presented in this study are included in the article/Supplementary Material.

## Supplementary Materials

The following supporting information can be downloaded at: https://www.mdpi.com/article/10.3390/ijms27177914/s1.
