# Supplementary material for: Hexaamminecobalt(III) Chromate and Dichromate as Precursors of Nanoscale Spinels Co(Co1−xCrx)2O4 (x = 0.75; 0.90; 1)
Source: Int J Mol Sci. 2026 Sep 4;27(17):7914. doi: 10.3390/ijms27177914 (PMC13566659; doi:10.3390/ijms27177914)
Supplement: Supplementary file 1 [file ijms-27-07914-s001.zip › ijms-4524461-supplementary.pdf]

Supplementary materials for

Hexaamminecobalt(III) chromate and dichromate as precursors of nanosized spinels $Co(Co_{1-x}Cr_x)_2O_4$ (x= 0.75; 0.90; 1)

Filatov E.Yu.[1,*], Tarasova P.V.[1], Sinitsa V.I.[1], Kostin G.A.[1], Kuratieva N.V.[1], Plyusnin P.E.[1], Korenev S.V.[1]

[1]Nikolaev Institute of Inorganic Chemistry SB RAS, Novosibirsk, Russia
[*]e-mail: decan@niic.nsc.ru

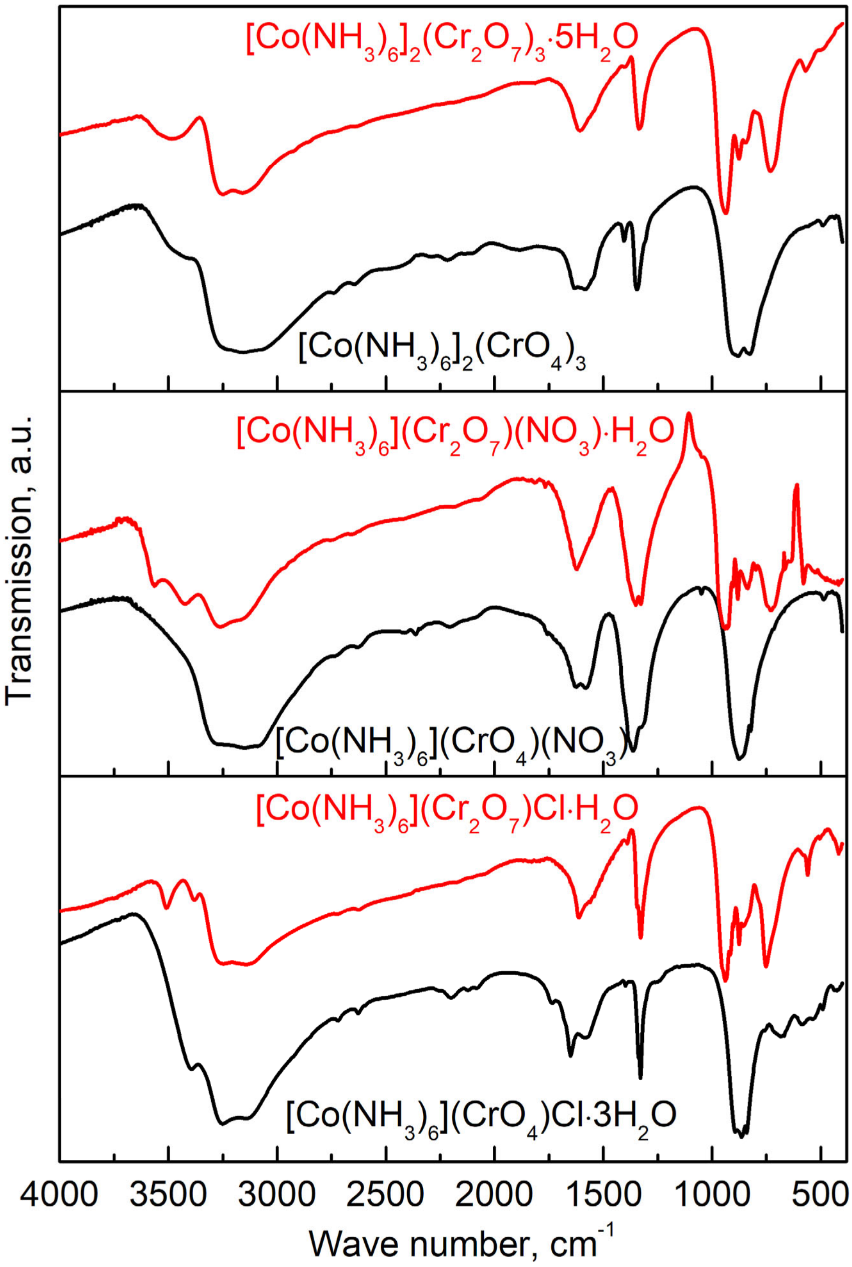

Figure S1. IR spectra of synthesized compounds.

Table S1. Infrared data for salts containing the $[Co(NH_3)_6]^{3+}$ cation.

	ν(O–H)	ν_{as}(N–H)	ν_s(N–H)	δ_d(N–H)	δ_s(N–H)	ν(N–O)	ν(Cr–O)	ρ_r(N–H)	ν(Cr–O–Cr)
$[Co(NH_3)_6](CrO_4)Cl\cdot 3H_2O$	3400	3250	3152	1650	1330		860		
$[Co(NH_3)_6](CrO_4)NO_3$		3160		1600	1360		870		
$[Co(NH_3)_6]_2(CrO_4)_3$		3150		1600	1350		890	825	
$[Co(NH_3)_6](Cr_2O_7)Cl\cdot H_2O$	3510<br>3385	3255	3125	1610	1330		940	875	750
$[Co(NH_3)_6](Cr_2O_7)(NO_3)\cdot H_2O$	3565<br>3430	3265	3165	1620	1350	835	930	880	730
$[Co(NH_3)_6]_2(Cr_2O_7)_3\cdot 5H_2O$	3485	3250	3160	1605	1335		940	875	730

Table S2. Donor-Acceptor distances for the H-bonds in compounds $[Co(NH_3)_6](CrO_4)(NO_3)$ (**II**).

Number	Atom1	Atom2	"Symm. op. 1"	"Symm. op. 2"	Length, Å
1	N11	O2	x,y,z	x,y,z	2.975
2	N13	O2	x,y,z	x,y,z	2.941
3	N12	O4	2-x,1/2-y,z	x,y,z	2.963
4	N11	O4	x,y,z	2-x,-y,-z	2.967
5	N14	O1	x,y,z	2-x,-y,-z	2.923
6	N12	O1	2-x,1/2-y,z	2-x,-y,-z	2.946
7	N11	O1N	x,y,z	1.5-x,y,-z	3.031
8	N21	O1	x,y,z	x,y,z	3.037
9	N21	O2	x,y,z	x,y,z	3.013
10	N23	O2	x,y,z	x,y,z	3.095
11	N23	O3	x,y,z	x,y,z	3.077
12	N22	O1	3/4+y,-3/4+x,1/4-z	x,y,z	2.970
13	N22	O3	x,y,z	-1/2+x,-y,z	2.863
14	N24	O3	x,y,z	-1/2+x,-y,z	2.950
15	N21	O2N	x,y,z	x,y,z	3.073
16	N24	O3N	x,y,z	1/4+y,3/4-x,1/4-z	2.948
17	N21	O3N	3/4+y,-3/4+x,1/4-z	1/4+y,3/4-x,1/4-z	3.093

Table S3. Donor-Acceptor distances for the H-bonds in compounds $[Co(NH_3)_6]_2(CrO_4)_3 \cdot 5H_2O$ (**III**).

Number	Atom1	Atom2	"Symm. op. 1"	"Symm. op. 2"	Length, Å
1	N13	O11	x,y,z	x,1+y,z	2.915
2	N16	O13	x,y,z	1/2-x,1/2+y,1/2-z	2.995
3	N14	O24	x,y,z	-1/2+x,1/2+y,z	2.920
4	N15	O24	x,y,z	-1/2+x,1/2+y,z	3.063
5	N12	O21	x,y,z	1/2-x,1.5-y,-z	2.940
6	N13	O21	x,y,z	1/2-x,1.5-y,-z	2.952
7	N11	O32	x,y,z	-x,y,1/2-z	2.868
8	N14	O32	x,y,z	-x,y,1/2-z	3.055
9	N15	O33	x,y,z	-x,y,1/2-z	3.092
10	N12	O31	x,y,z	x,1-y,-1/2+z	2.951
11	N12	O2W	x,y,z	x,y,z	2.897
12	N12	O3W	x,y,z	x,y,z	2.916
13	N16	O3W	x,y,z	x,y,z	2.981
14	N14	O5W	x,y,z	x,y,z	2.908
15	N21	O14	x,y,z	x,y,z	2.935
16	N23	O14	x,y,z	x,y,z	3.000
17	N22	O21	x,y,z	x,y,z	2.934
18	N21	O21	1/2-x,1/2-y,-z	x,y,z	3.025
19	N23	O34	x,y,z	1/2-x,-1/2+y,1/2-z	2.978
20	N22	O33	1/2-x,1/2-y,-z	1/2-x,-1/2+y,1/2-z	3.043
21	N22	O1W	x,y,z	x,y,z	2.976
22	N23	O1W	x,y,z	x,y,z	2.861
23	N33	O12	x,y,z	x,y,z	2.910
24	N34	O12	x,y,z	x,y,z	2.894
25	N31	O22	x,y,z	-1/2+x,-1/2+y,z	2.913
26	N33	O23	x,y,z	-1/2+x,-1/2+y,z	2.984
27	N34	O22	-x,y,1/2-z	-1/2+x,-1/2+y,z	3.081
28	N32	O32	x,y,z	x,y,z	3.087

29	N34	O31	x,y,z	x,y,z	2.961
30	N33	O32	-x,y,1/2-z	x,y,z	3.057
31	N32	O4W	x,y,z	x,y,z	3.095
32	O12	O1W	x,y,z	x,y,z	2.694
33	O13	O1W	x,y,z	1/2-x,-1/2+y,1/2-z	2.707
34	O13	O2W	x,y,z	1/2-x,-1/2+y,1/2-z	2.782
35	O14	O3W	x,y,z	x,-1+y,z	2.663
36	O13	O4W	x,y,z	1/2-x,-1/2+y,1/2-z	2.877
37	O21	O2W	x,y,z	x,y,z	2.919
38	O22	O2W	x,y,z	x,y,z	3.056
39	O22	O5W	x,y,z	1/2-x,-1/2+y,1/2-z	2.879
40	O31	O3W	x,y,z	1/2-x,-1/2+y,1/2-z	2.819
41	O34	O4W	x,y,z	x,y,z	2.767
42	O34	O5W	x,y,z	x,y,z	3.050

Table S4. Donor-Acceptor distances for the H-bonds in compounds $[Co(NH_3)_6](Cr_2O_7)(NO_3)\cdot H_2O$ (**V**).

Number	Atom1	Atom2	"Symm. op. 1"	"Symm. op. 2"	Length, Å
1	N11	O3	x,y,z	-1+x,y,z	3.000
2	N13	O1	x,y,z	-1+x,y,z	3.098
3	N12	O3	x,y,z	-1+x,1+y,z	2.963
4	N13	O2	x,y,z	-1+x,1+y,z	2.977
5	N11	O6	x,y,z	x,y,z	2.994
6	N12	O1N	x,y,z	x,y,z	3.011
7	N13	O2N	x,y,z	x,y,z	2.927
8	N22	O4	x,y,z	-1+x,y,z	3.024
9	N23	O7	-x,-y,1-z	-1+x,y,z	2.982
10	N21	O7	x,y,z	x,y,z	3.091
11	N23	O3N	x,y,z	x,-1+y,z	2.994
12	N21	O3N	-x,-y,1-z	x,-1+y,z	2.969
13	N21	O1W	x,y,z	x,-1+y,z	3.063
14	O6	O1W	x,y,z	x,-1+y,z	3.035
15	O5	O1W	x,y,z	x,y,z	2.809

Table S5. Donor-Acceptor distances for the H-bonds in compounds $[Co(NH_3)_6]_2(Cr_2O_7)_3 \cdot 5H_2O$ with space group $P2_1/n$ (**VI_1**).

Number	Atom1	Atom2	"Symm. op. 1"	"Symm. op. 2"	Length, Å
1	N11	O11	x,y,z	x,y,z	3.010
2	N12	O11	x,y,z	x,y,z	3.077
3	N15	O15	x,y,z	x,y,z	2.970
4	N15	O13	x,y,z	1/2+x,1/2-y,1/2+z	3.079
5	N12	O21	x,y,z	x,y,z	2.993
6	N13	O25	x,y,z	x,y,z	3.017
7	N15	O25	x,y,z	x,y,z	2.949
8	N11	O22	x,y,z	1/2-x,-1/2+y,1/2-z	3.037
9	N11	O26	x,y,z	-1/2+x,1/2-y,-1/2+z	2.916
10	N16	O26	x,y,z	-1/2+x,1/2-y,-1/2+z	2.991
11	N12	O36	x,y,z	-1+x,y,z	2.969
12	N12	O32	x,y,z	-1/2+x,1/2-y,1/2+z	3.069
13	N14	O2W	x,y,z	x,y,z	3.008
14	N13	O4WA	x,y,z	-1/2+x,1/2-y,1/2+z	2.887
15	N16	O4WA	x,y,z	-1/2+x,1/2-y,1/2+z	3.069
16	N13	O5WA	x,y,z	1-x,-y,1-z	2.962
17	N13	O5WB	x,y,z	1-x,-y,1-z	2.976
18	N22	O16	x,y,z	x,y,z	3.050
19	N21	O13	x,y,z	1/2+x,1/2-y,1/2+z	2.970
20	N24	O17	x,y,z	1/2+x,1/2-y,1/2+z	2.933
21	N22	O27	x,y,z	x,y,z	3.021
22	N25	O27	x,y,z	x,y,z	2.938
23	N22	O24	x,y,z	1-x,1-y,1-z	2.958
24	N25	O22	x,y,z	1-x,1-y,1-z	3.076
25	N21	O31	x,y,z	x,y,z	3.078
26	N21	O35	x,y,z	x,y,z	3.073
27	N24	O36	x,y,z	x,y,z	2.938
28	N26	O31	x,y,z	x,y,z	3.013

29	N23	O34	x,y,z	1.5-x,1/2+y,1/2-z	3.013
30	N26	O37	x,y,z	1.5-x,1/2+y,1/2-z	2.887
31	N24	O32	x,y,z	1/2+x,1/2-y,1/2+z	3.083
32	N23	O1W	x,y,z	1+x,y,z	2.949
33	N26	O3W	x,y,z	x,y,z	3.020
34	O13	O2W	x,y,z	-1/2+x,1/2-y,-1/2+z	3.018
35	O12	O3W	x,y,z	-1+x,y,z	2.934
36	O16	O4WA	x,y,z	x,y,z	3.013
37	O16	O4WB	x,y,z	x,y,z	2.978
38	O17	O4WA	x,y,z	x,y,z	2.946
39	O12	O5WA	x,y,z	1/2-x,1/2+y,1/2-z	3.021
40	O14	O5WB	x,y,z	-1/2+x,1/2-y,-1/2+z	2.988
41	O22	O1W	x,y,z	x,y,z	2.839
42	O22	O2W	x,y,z	1/2-x,1/2+y,1/2-z	3.096
43	O25	O3W	x,y,z	-1/2+x,1/2-y,1/2+z	2.994
44	O33	O2W	x,y,z	x,y,z	2.901
45	O35	O2W	x,y,z	x,y,z	2.946
46	O36	O3W	x,y,z	x,y,z	2.831
47	O35	O5WA	x,y,z	x,y,z	2.949
48	O35	O5WB	x,y,z	x,y,z	2.863
49	O1W	O3W	x,y,z	-1+x,y,z	2.827
50	O4WB	O5WB	x,y,z	1.5-x,1/2+y,1/2-z	2.886

Table S6. Donor-Acceptor distances for the H-bonds in compounds $[Co(NH_3)_6]_2(Cr_2O_7)_3\cdot 5H_2O$ with space group $P2_1/c$ (**VI$_2$**).

Number	Atom1	Atom2	"Symm. op. 1"	"Symm. op. 2"	Length, Å
1	N11	O11	x,y,z	x,y,z	3.091
2	N14	O15	x,y,z	x,y,z	2.995
3	N16	O15	x,y,z	x,y,z	3.055
4	N12	O17	x,y,z	x,1/2-y,-1/2+z	3.058
5	N14	O26	x,y,z	x,-1+y,z	3.082
6	N15	O26	x,y,z	x,-1+y,z	2.949
7	N11	O23	x,y,z	x,1/2-y,-1/2+z	2.894
8	N12	O23	x,y,z	x,1/2-y,-1/2+z	2.957
9	N13	O35	x,y,z	x,-1+y,z	3.038
10	N14	O35	x,y,z	x,-1+y,z	2.930
11	N13	O33	x,y,z	1-x,-1/2+y,1/2-z	2.970
12	N13	O36	x,y,z	1-x,1-y,-z	2.994
13	N11	O1W	x,y,z	-x,-1/2+y,1/2-z	2.944
14	N14	O1W	x,y,z	-x,-1/2+y,1/2-z	3.015
15	N16	O5W	x,y,z	1-x,-1/2+y,1/2-z	3.067
16	N21	O12	x,y,z	x,y,z	3.075
17	N24	O16	x,y,z	x,y,z	2.953
18	N25	O12	x,y,z	x,y,z	3.069
19	N25	O16	x,y,z	x,y,z	2.941
20	N21	O13	x,y,z	-x,1-y,-z	3.070
21	N26	O13	x,y,z	-x,1-y,-z	3.032
22	N25	O17	x,y,z	x,1/2-y,-1/2+z	3.094
23	N21	O21	x,y,z	x,y,z	2.999
24	N24	O25	x,y,z	x,y,z	2.976
25	N21	O22	x,y,z	-x,1-y,-z	2.969
26	N22	O22	x,y,z	-x,1-y,-z	3.039
27	N25	O22	x,y,z	-x,1-y,-z	3.025
28	N22	O27	x,y,z	x,1.5-y,-1/2+z	2.944

29	N24	O31	x,y,z	x,y,z	3.059
30	N26	O31	x,y,z	x,y,z	3.040
31	N23	O37	x,y,z	1-x,-1/2+y,1/2-z	3.005
32	N22	O32	x,y,z	1-x,1-y,-z	3.091
33	N23	O32	x,y,z	1-x,1-y,-z	3.017
34	N26	O4W	x,y,z	x,1.5-y,-1/2+z	3.063
35	N25	O5W	x,y,z	1-x,-1/2+y,1/2-z	3.076
36	O15	O1W	x,y,z	-x,-1/2+y,1/2-z	2.928
37	O14	O2W	x,y,z	x,y,z	2.832
38	O11	O2W	x,y,z	x,1/2-y,-1/2+z	3.032
39	O17	O5W	x,y,z	1-x,1-y,1-z	3.003
40	O24	O2W	x,y,z	-x,1/2+y,1/2-z	2.874
41	O25	O3W	x,y,z	x,y,z	2.792
42	O33	O3W	x,y,z	x,y,z	2.957
43	O37	O4W	x,y,z	x,y,z	2.886
44	O34	O5W	x,y,z	x,1.5-y,-1/2+z	2.855
45	O1W	O2W	x,y,z	x,y,z	2.790
46	O3W	O4W	x,y,z	x,y,z	2.914

Table S7. Hirshfeld surfaces and 2D fingerprint plots for structurally independent cations in the complex salts.

**Hirshfeld surfaces**	**2D plot (all interactions)**	**2D plot (H…O interactions)**
$[Co(NH_3)_6](CrO_4)(NO_3)$ **II**		
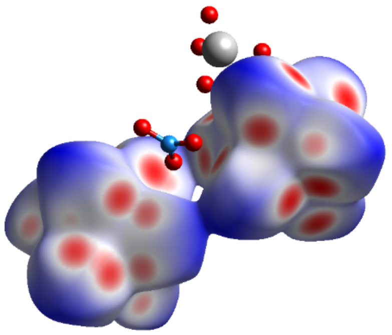	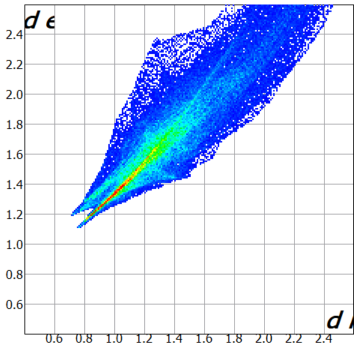	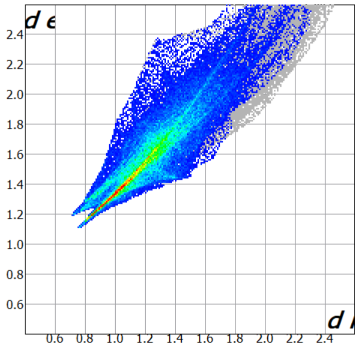
$[Co(NH_3)_6]_2(CrO_4)_3·5H_2O$ **III**		
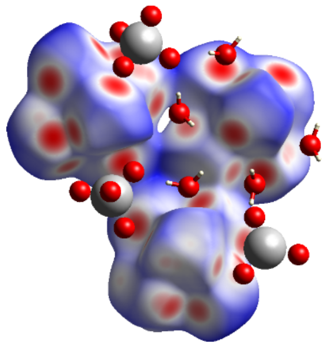	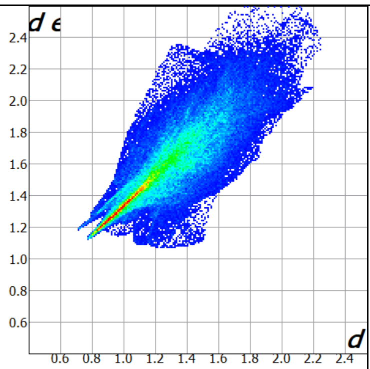	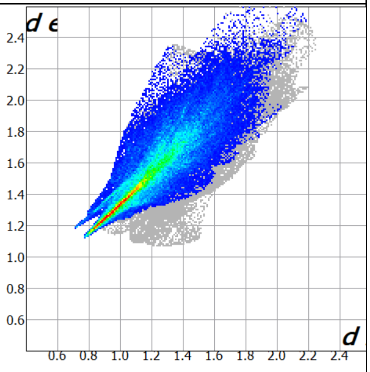
$[Co(NH_3)_6](Cr_2O_7)(NO_3)·H_2O$ **V**		
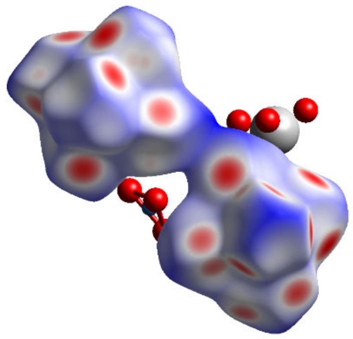	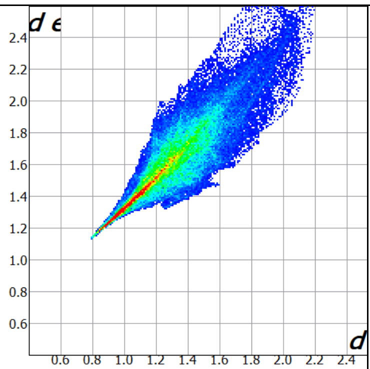	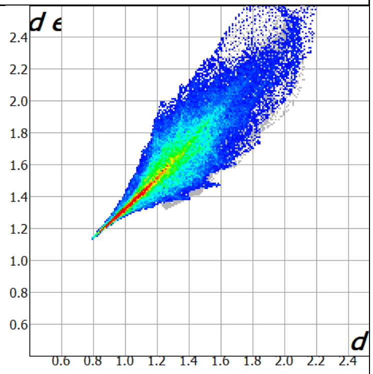
$[Co(NH_3)_6]_2(Cr_2O_7)_3·5H_2O$ (sp. gr. $P2_1/n$) **VI₁**		
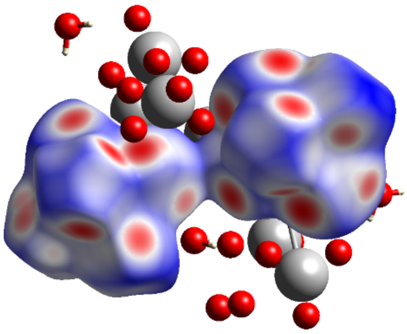	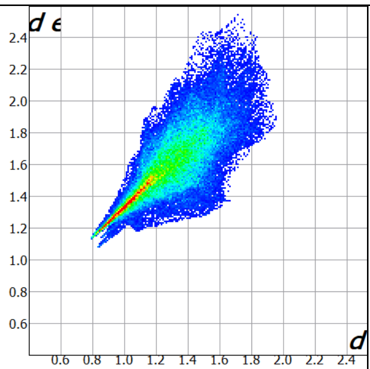	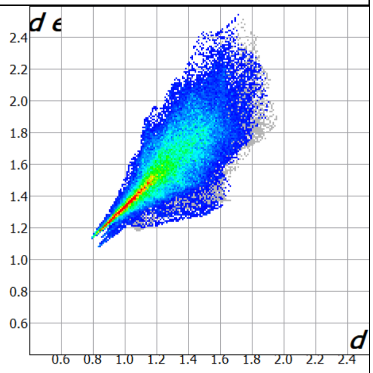

$[Co(NH_3)_6]_2(Cr_2O_7)_3 \cdot 5H_2O$ (sp. gr. $P2_1/c$) **VI2**

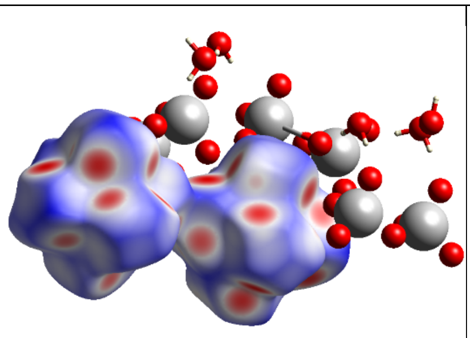

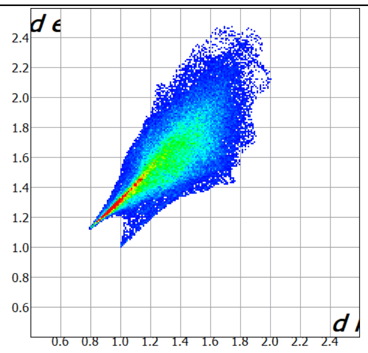

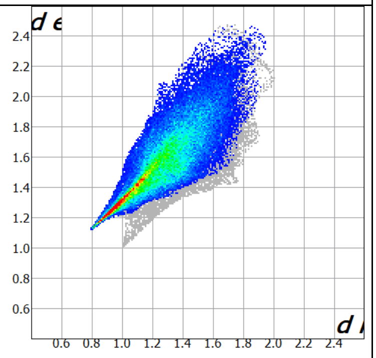

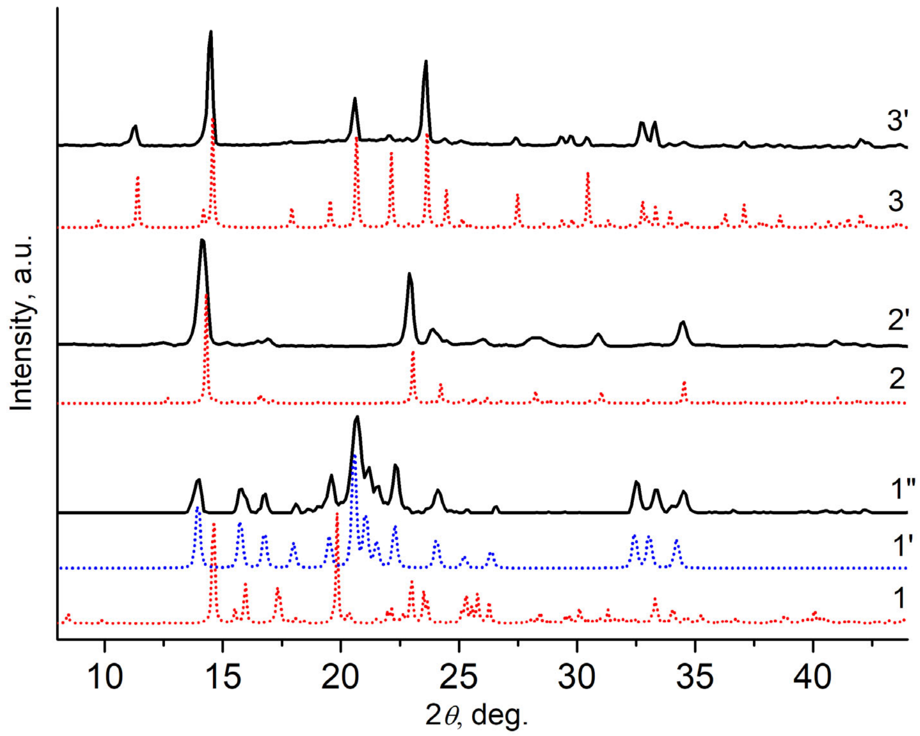

Figure S2. XRD pattern obtained from the single-crystal data (**1, 2, 3**) as compared with the powder XRD patterns for $[Co(NH_3)_6]_2(CrO_4)_3$ (**1"**), $[Co(NH_3)_6](CrO_4)(NO_3)$ (**2'**) and $[Co(NH_3)_6](CrO_4)Cl\cdot 3H_2O$ (**3'**). **1'** – powder XRD pattern for $[Cr(NH_3)_6]_2(CrO_4)_3$ (PDF #28-359); **1** – XRD pattern obtained from the single-crystal data for $[Co(NH_3)_6]_2(CrO_4)_3\cdot 5H_2O$.

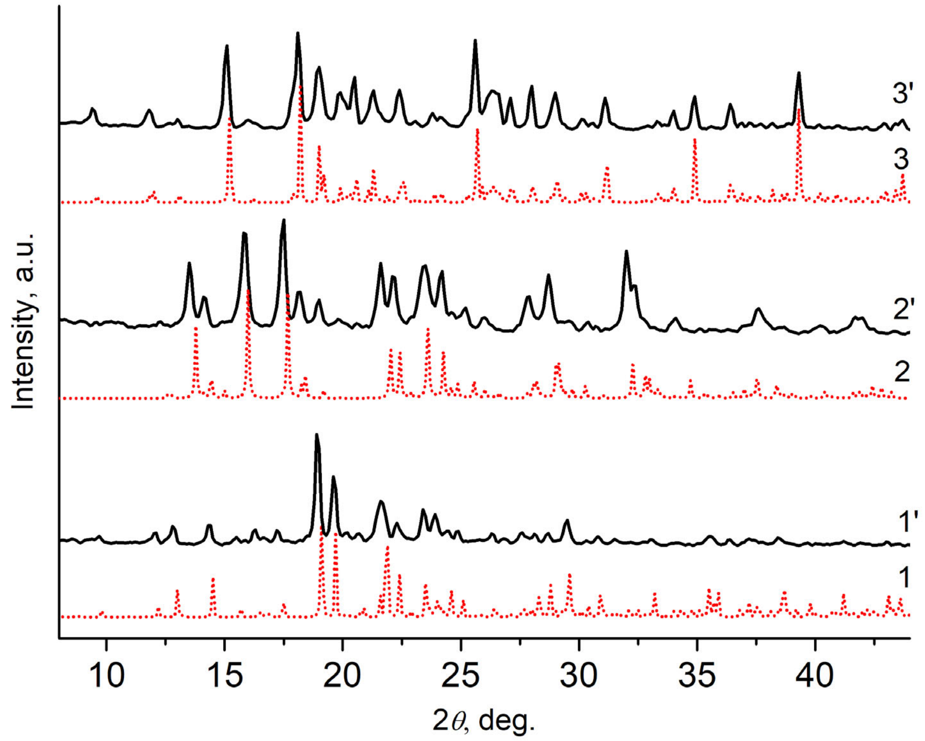

Figure S3. XRD pattern obtained from the single-crystal data (**1, 2, 3**) as compared with the powder XRD patterns for $[Co(NH_3)_6]_2(Cr_2O_7)_3\cdot 5H_2O$ (**1'**), $[Co(NH_3)_6](Cr_2O_7)(NO_3)\cdot H_2O$ (**2'**) and $[Co(NH_3)_6](Cr_2O_7)Cl\cdot H_2O$ (**3'**).

Table S8. Diffraction patterns of thermolysis products in an inert atmosphere at 600 ºC of the synthesized compounds and their simulation of the corresponding phases using the Rietveld method. The structural models/reference phases used in the refinements: $CoCr_2O_4$ (ICSD #69503), Cr_2O_3 (ICSD #250078), $Co_2(OH)_3Cl$ (ICSD #155534). The peak profile was described by the Pseudo-Voigt2 function.

Initial compound: $[Co(NH_3)_6]_2(CrO_4)_3$<br>Product(s): $Co_{1.2}Cr_{1.8}O_4$ (100 wt.%, sp. gr. *Fd*-3*m*, *a* = 8.302(5) Å).<br>*Rwp* = 4.6 %, *Rp* = 3.5 %, *GOF* = 1.0 %.<br>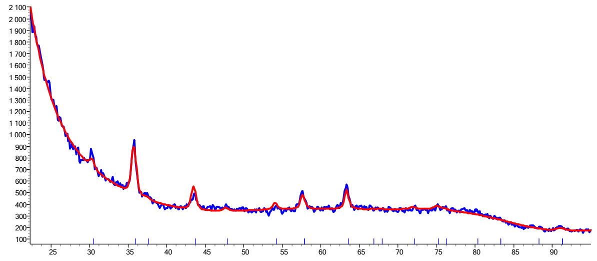	Initial compound: $[Co(NH_3)_6]_2(Cr_2O_7)_3$<br>Product(s): $CoCr_2O_4$ (78(3) wt.%, sp. gr. *Fd*-3*m*, *a* = 8.324(4) Å) and Cr_2O_3 (22(3) wt.%, sp. gr. *R*-3*c*, *a* = 4.958(4) Å, *c* = 13.593(6) Å).<br>*Rwp* = 10.4 %, *Rp* = 7.9 %, *GOF* = 2.6 %.<br>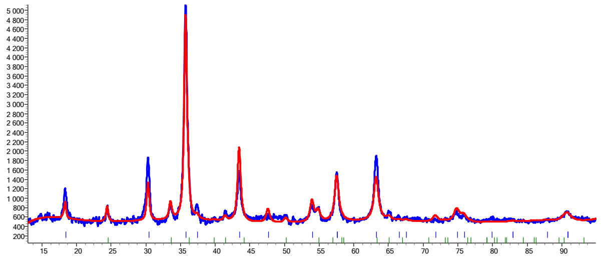
Initial compound: $[Co(NH_3)_6](CrO_4)(NO_3)$<br>Product(s): $Co_{1.5}Cr_{1.5}O_4$, (100 wt.%, sp. gr. *Fd*-3*m*, *a* = 8.270(5) Å).<br>*Rwp* = 4.3 %, *Rp* = 3.5 %, *GOF* = 1.0 %.<br>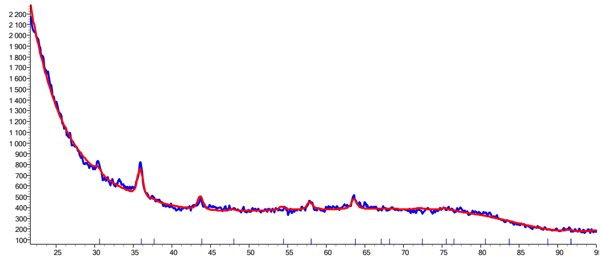	Initial compound: $[Co(NH_3)_6](Cr_2O_7)(NO_3)$<br>Product(s):): $CoCr_2O_4$ (100 wt.%, sp. gr. *Fd*-3*m*, *a* = 8.324(4) Å).<br>*Rwp* = 7.3 %, *Rp* = 5.6 %, *GOF* = 1.7 %.<br>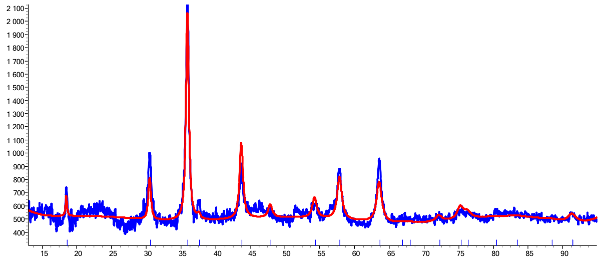
Initial compound: $[Co(NH_3)_6](CrO_4)Cl$<br>Product(s): $CoCr_2O_4$ (74(5) wt.%, sp. gr. *Fd*-3*m*, *a* = 8.324(5) Å) and $Co_2(OH)_3Cl$ (26(5) wt.%, sp. gr. *R*-3*mH*, *a* = 6.842(5) Å, *c* = 14.508(7) Å).<br>*Rwp* = 10.4 %, *Rp* = 8.3 %, *GOF* = 2.8 %.<br>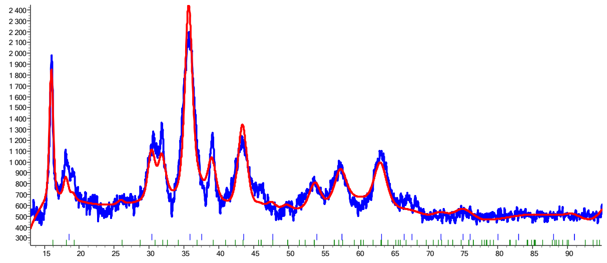	Initial compound: $[Co(NH_3)_6](Cr_2O_7)Cl$<br>Product(s): $CoCr_2O_4$ (100 wt.%, sp. gr. *Fd*-3*m*, *a* = 8.326(4) Å).<br>*Rwp* = 10.5 %, *Rp* = 8.6 %, *GOF* = 3.0 %.<br>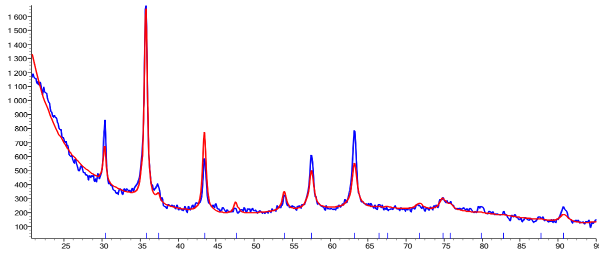

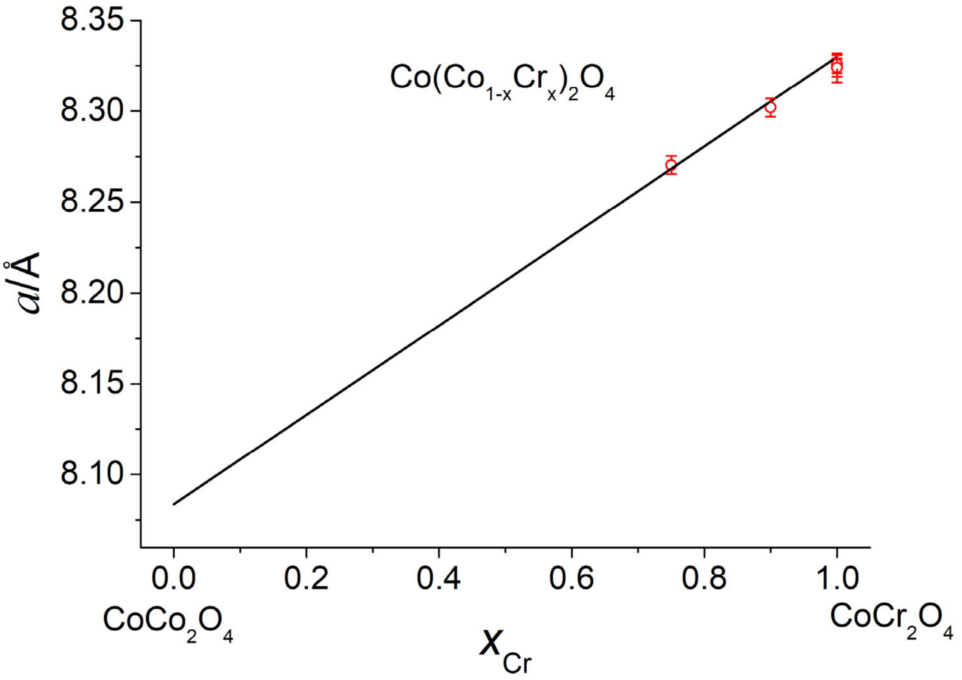

Figure S4. Calibration curve of the unit cell parameter of spinel $Co(Co_{1-x}Cr_x)O_4$ versus chromium content (x_{Cr}). The calibration equation: $a = 8.084 + 0.246 \cdot x_{Cr}$. The unit cell parameters of the products obtained in this study are shown in red dots.

Table S9. Crystal data and conditions of the single crystal XRD experiment for $[Co(NH_3)_6]_2(CrO_4)_3 \cdot 5H_2O$ and $[Co(NH_3)_6]_2(Cr_2O_7)_3 \cdot 5H_2O$.

Empirical formula	Co2H46Cr3N12O17	Co2H46Cr6N12O26	Co2H46Cr6N12O26
Identification code	*iks014*	*iks002*	*iks006*
CCDC number	*2533412*	*2533415*	*2533414*
Formula weight	*760.35*	*1060.35*	*1060.35*
Temperature/K	*150(2)*	*293(2)*	*293(2)*
Crystal system	*monoclinic*	*monoclinic*	*monoclinic*
Space group	*C2/c*	$P2_1/c$	$P2_1/n$
a/Å	*21.4836(6)*	*15.5295(4)*	*12.8963(6)*
b/Å	*11.8653(3)*	*14.8497(4)*	*18.0157(9)*
c/Å	*21.3035(5)*	*15.3209(4)*	*15.2677(7)*
α/°	*90*	*90*	*90*
β/°	*101.0200(10)*	*106.3010(10)*	*108.689(2)*
γ/°	*90*	*90*	*90*
Volume/Å^3	*5330.3(2)*	*3391.10(16)*	*3360.2(3)*
Z	*8*	*4*	*4*
Pcalc/g/cm^3	*1.895*	*2.077*	*2.096*
μ/mm^{-1}	*2.488*	*2.903*	*2.929*
F(000)	*3136*	*2144*	*2144*
Crystal size/mm^3	*0.30 × 0.20 × 0.17*	*0.15 × 0.15 × 0.10*	*0.20 × 0.10 × 0.06*
Radiation	*MoKα (λ = 0.71073)*	*MoKα (λ = 0.71073)*	*MoKα (λ = 0.71073)*
2Θ range for data collection/°	1.931 to 30.080	2.594 to 27.479	1.805 to 27.497
Index ranges	*-23<=h<=30, -16<=k<=16, -29<=l<=29*	*-20<=h<=12, -19<=k<=16, -18<=l<=19*	*-16<=h<=16, -21<=k<=23, -19<=l<=19*
Reflections collected	*20874*	*18034*	*19659*
Independent reflections	*7788 [R(int) = 0.0251]*	*7708 [Rint = 0.0392]*	*7688 [Rint = 0.0345]*
Data/restraints/parameters	*7788 / 12 / 364*	*7708 / 17 / 458*	*7688 / 17 / 463*
Goodness-of-fit on F^2	*1.104*	*1.039*	*1.017*

Final R indexes [I>=2σ (I)]	*R1 = 0.0408, wR2 = 0.0916*	*R1 = 0.0484, wR2 = 0.1125*	*R1 = 0.0336, wR2 = 0.0786*
Final R indexes [all data]	*R1 = 0.0483, wR2 = 0.0945*	*R1 = 0.0757, wR2 = 0.1222*	*R1 = 0.0504, wR2 = 0.0833*
Largest diff. peak/hole / e Å^{-3}	*1.087 / -0.902*	*0.675 / -0.635*	*0.469 / -0.460*

Table S10. Crystal data and conditions of the single crystal XRD experiment for $[Co(NH_3)_6](CrO_4)(NO_3)$ and $[Co(NH_3)_6](Cr_2O_7)(NO_3)\cdot H_2O$.

Empirical formula	*CoCrH18N7O7*	*CoCr2H20N7O11*
Identification code	*pol054*	*pol014*
CCDC number	*2533411*	*2533413*
Formula weight	*339.14*	*457.16*
Temperature/K	*293(2)*	*150(2)*
Crystal system	*tetragonal*	*triclinic*
Space group	*$I4_1/acd$*	*P-1*
a/Å	*14.6807(4)*	*7.5304(2)*
b/Å	*14.6807(4)*	*7.62210(10)*
c/Å	*46.0730(14)*	*13.9086(3)*
α/°	*90*	*85.7400(10)*
β/°	*90*	*86.2370(10)*
γ/°	*90*	*67.7350(10)*
Volume/Å^3	*9929.8(6)*	*736.15(3)*
Z	*32*	*2*
Pcalc/g/cm^3	*1.815*	*2.062*
μ/mm^{-1}	*2.245*	*2.629*
F(000)	*5568*	*464*
Crystal size/mm^3	*0.20 × 0.15 × 0.07*	*0.26 × 0.16 × 0.11*
Radiation	*MoKα (λ = 0.71073)*	*MoKα (λ = 0.71073)*
2Θ range for data collection/°	*1.768 to 27.549*	*2.892 to 27.148*
Index ranges	*-18<=h<=19, -12<=k<=19, -59<=l<=59*	*-9<=h<=9, -9<=k<=9, -17<=l<=17*
Reflections collected	*35210*	*6548*
Independent reflections	*2860 [R(int) = 0.0508]*	*3223 [R(int) = 0.0255]*
Data/restraints/parameters	*2860 / 0 / 156*	*3223 / 2 / 207*
Goodness-of-fit on F^2	*1.068*	*1.039*

Final R indexes [I>=2σ (I)]	R1 = 0.0338, wR2 = 0.0868	R1 = 0.0213, wR2 = 0.0604
Final R indexes [all data]	R1 = 0.0490, wR2 = 0.0934	R1 = 0.0226, wR2 = 0.0611
Largest diff. peak/hole / e $Å^{-3}$	1.668 / -0.297	0.343 / -0.361
